# Serum-Dependent Selective Expression of EhTMKB1-9, a Member of *Entamoeba histolytica* B1 Family of Transmembrane Kinases

**DOI:** 10.1371/journal.ppat.1000929

**Published:** 2010-06-03

**Authors:** Shiteshu Shrimal, Sudha Bhattacharya, Alok Bhattacharya

**Affiliations:** 1 School of Life Sciences, Jawaharlal Nehru University, New Delhi, India; 2 School of Environmental Sciences, Jawaharlal Nehru University, New Delhi, India; 3 School of Information Technology, Jawaharlal Nehru University, New Delhi, India; University of California Los Angeles, United States of America

## Abstract

*Entamoeba histolytica* transmembrane kinases (EhTMKs) can be grouped into six distinct families on the basis of motifs and sequences. Analysis of the *E. histolytica* genome revealed the presence of 35 EhTMKB1 members on the basis of sequence identity (≥95%). Only six homologs were full length containing an extracellular domain, a transmembrane segment and an intracellular kinase domain. Reverse transcription followed by polymerase chain reaction (RT-PCR) of the kinase domain was used to generate a library of expressed sequences. Sequencing of randomly picked clones from this library revealed that about 95% of the clones were identical with a single member, EhTMKB1-9, in proliferating cells. On serum starvation, the relative number of EhTMKB1-9 derived sequences decreased with concomitant increase in the sequences derived from another member, EhTMKB1-18. The change in their relative expression was quantified by real time PCR. Northern analysis and RNase protection assay were used to study the temporal nature of EhTMKB1-9 expression after serum replenishment of starved cells. The results showed that the expression of EhTMKB1-9 was sinusoidal. Specific transcriptional induction of EhTMKB1-9 upon serum replenishment was further confirmed by reporter gene (luciferase) expression and the upstream sequence responsible for serum responsiveness was identified. EhTMKB1-9 is one of the first examples of an inducible gene in *Entamoeba*. The protein encoded by this member was functionally characterized. The recombinant kinase domain of EhTMKB1-9 displayed protein kinase activity. It is likely to have dual specificity as judged from its sensitivity to different kinase inhibitors. Immuno-localization showed EhTMKB1-9 to be a surface protein which decreased on serum starvation and got relocalized on serum replenishment. Cell lines expressing either EhTMKB1-9 without kinase domain, or EhTMKB1-9 antisense RNA, showed decreased cellular proliferation and target cell killing. Our results suggest that *E. histolytica* TMKs of B1 family are functional kinases likely to be involved in serum response and cellular proliferation.

## Author Summary

The presence of a vast array of putative transmembrane kinase genes suggests an extensive network of signaling systems in *E. histolytica*, particularly the ability to perceive signals from the extracellular environment and transduce these intracellularly. However, it has been very difficult to work with these molecules due to the presence of a large number of homologs. It is also not clear if these molecules are indeed protein kinases, as no kinase activity has yet been shown associated with these molecules. In this report, we show that EhTMKB1-9 is a protein kinase and it is one of the early serum-induced genes. It is a predominant EhTMKB1 molecule that is expressed in proliferating cells and its expression is modulated by serum. Cells containing a reduced level of EhTMKB1-9 or high level of a mutant protein result in decreased proliferation, target cell killing and adherence. The results presented in this report suggest that EhTMKB1-9 is an important signaling molecule likely to be involved in *E. histolytica* proliferation and virulence. We have also identified a serum starvation induced response where expression of EhTMKB1-18 was found to be induced.

## Introduction

Transmembrane kinases (TMKs) play a major role in a number of essential processes in almost all eukaryotic cells and generally contain an extracellular domain, a transmembrane domain and an intracellular serine/threonine or tyrosine kinase domain. They are essentially involved in sensing and transducing extracellular signals to the appropriate sub cellular machinery. Mammalian TMKs, such as epidermal growth factor receptor (EGFR) have been studied extensively. EGFR undergoes EGF-induced dimerization that leads to activation of the intracellular kinase domain [1] and consequently the MAPK pathway is turned on, resulting in cellular proliferation. Genome analysis has shown that TMKs are present in abundance in plants, for example 610 in *Arabidopsis thaliana* [2]. However, the functional role of only a few of these has been elucidated [3], [4]. Generally the number of putative TMKs declines in organisms with decreasing complexity, for example there are 70 TMKs in humans [5]–[8], 43 in *Caenorhabditis elegans* [9] and 12 TMKs in *Dictyostelium discoideum* [10].

Analysis of the draft genome sequence of the protistan parasite *Entamoeba histolytica* indicated 90 putative TMKs that bear striking resemblance with the intermediate subunit of amoebic Gal/GalNAc lectin [11], [12]. All EhTMKs contain an N-terminal signal peptide, a predicted extracellular domain and a single transmembrane helix followed by a cytosolic tyrosine kinase-like domain. Beck *et al.*, have grouped these genes into six distinct families (A to F) based on motifs in the extracellular and kinase domains [12]. The extracellular domain of transmembrane protein kinases contains CXXC-rich repeats which are also found in the intermediate subunit (Igl) of the Gal/GalNAc lectin and *Giardia lamblia* variant-specific surface proteins. Spotted oligoarrays and real-time PCR showed that EhTMKs belonging to different families are expressed in *E. histolytica* cells and that the level of expression of individual TMKs differed significantly [12]. Sequence analysis of EhTMKs demonstrated similarities to both serine/threonine and tyrosine kinases. The closest homolog of the *E. histolytica* TMK kinase domain is a cytoplasmic dual-specificity kinase, SplA, from *D. discoideum* [13]. However, none of the putative extracellular ligand binding domains of EhTMKs showed any significant similarity with that of known heterologous TMKs.

The first evidence that suggested EhTMK to have a significant role in amebic biology came from studies on EhTMKB1 family. *E. histolytica* cells over expressing a truncated form of EhTMKB1-2 (B1.I.1, a full length member; Figure S1), showed defect in cellular proliferation [14]. Subsequently, EhTMKB3-96 (PATMK96) was shown to participate in erythro-phagocytosis and may, thus, be involved in pathogenesis [15]. However, these studies did not include a demonstration of functional activity of the kinase domain.

In this report, we show that of the 35 members of EhTMKB1 family, EhTMKB1-9 is the predominantly expressed member in proliferating *E. histolytica* cells. On serum starvation while EhTMKB1-9 transcription is down regulated, EhTMKB1-18 transcription goes up. The latter gene is unlikely to have any protein product. The expression of both EhTMKB1s is controlled by serum at the transcriptional level. The promoter region responsible for regulated expression of EhTMKB1-9 has been identified. We also show that serum contains a heat labile ligand which induces serum response and the kinase domain of EhTMKB1-9 has protein phosphorylating activity. Immuno-localization reveals EhTMKB1-9 to be a membrane protein. Over expression of the dominant negative mutant or blocking the expression of EhTMKB1-9 gene decreased the cellular growth and target cell killing indicating a significant role of the B1 family of transmembrane kinases in amoebic biology.

## Results

### EhTMKB1 family contains 35 members

**Figure 1:**
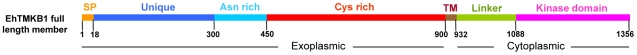
Schematic representation of EhTMKB1 full length member. The functional domain organization of a typical full length EhTMKB1 member based on amino acid composition is indicated schematically. Signal peptide sequence (SP), asparagine-rich region (Asn rich), cysteine-rich motifs (Cys rich), trans-membrane region (TM), linker and kinase domain.

*E. histolytica* TMKs have been classified into a number of different families based on sequence motifs [12]. The basic structural organization of a full length EhTMKB1 member is shown in Figure 1. It has an extracellular region of around 900 amino acids (containing an N-terminal signal peptide, a unique region, an asparagine rich region and a cysteine rich region), a transmembrane (900–932) domain and a cytosolic kinase domain (1088–1356). Sequence analysis of the first draft genome assembly of *E. histolytica* showed that the B1 family of EhTMKs contained 28 members [14]. Since the *E. histolytica* genome database has been updated, with improved assembly, we repeated the database search to update the list of members belonging to EhTMKB1 family. A comparison of the results from this analysis with the previous one is provided in explanation of Figure S1. A full-length EhTMKB1-1 member (XM_001913432) was used as query to carry out a BLAST search and 35 family members were identified on the basis of sequence identity (≥95%) at nucleotide level (Figure S1). The annotation was done on the basis of sequence identity, presence of domains, open reading frame (ORF) and the results of a coding region prediction algorithm “Genescan” [16], [17]. The identified sequences were dispersed on different contigs and only six of these were full length members. The majority of the EhTMKB1 members were truncated at their 5′- end, that is, they lacked significant sequence identity with the 5′- end of the query sequence. Some copies lacked the 3′- end of the query sequence while some lacked both the ends. Detailed information about EhTMKB1 members and accession numbers is in Table S1.The predicted gene structures of some of the copies extended beyond the conserved regions and are likely to have been acquired after the duplication event or the expansion process. Some of the examples are the N-terminal regions of the four members - EhTMKB1-7, 9, 10 and 11 (boxed region and shown by different colors in Figure S1). Further, it was not possible to predict the organization of some members as these copies are from one end of the respective contigs. In order to confirm the predicted organization of EhTMKB1 members, southern hybridization with probes specific to some of the members was performed and the results obtained were in agreement with predicted organization (Figure S2 and Table S2).

The genome of *Entamoeba dispar*, a non pathogenic sibling species of *E. histolytica*, was searched for the presence of EhTMKB1-like sequences. The search revealed 76 members with more than 85% identity at the nucleotide level (data not shown). Of these only four full length copies were identified. Although in the reptilian species, *Entamoeba invadens* it is difficult to predict if there are homologs of EhTMKB1s due to low level of sequence identity, it is likely that there may be four full length homologs with 60% sequence identity.

### Phylogenetic analysis of the *Entamoeba* TMKs

**Figure 2:**
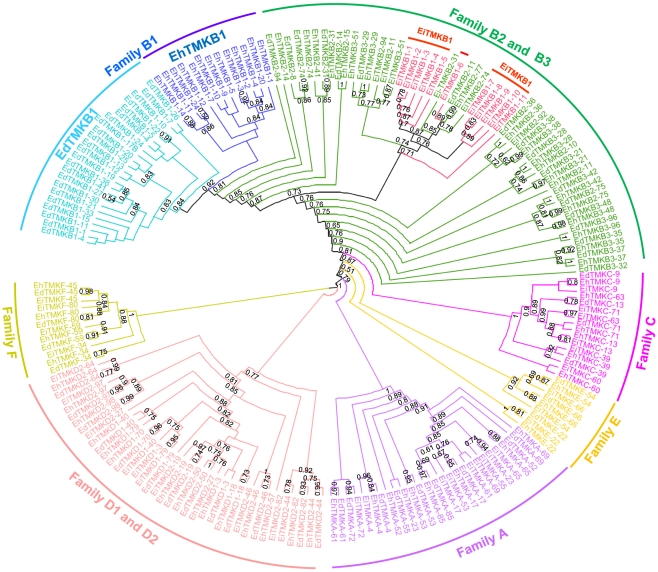
Phylogenetic analysis of *Entamoeba* transmembrane kinases based on sequence of kinase domain. The kinase domain sequence of the TMKs of *E. histolytica* (Eh), *E. dispar* (Ed) and *E. invadens* (Ei) belonging to different families (A to F) were extracted and aligned using MUSCLE. The automatic curation was performed by Gblock and tree building by PhyML. The significant bootstrap values above 50% are shown. Accession numbers of TMKs are listed in Table S3.

In order to determine the lineage of EhTMKB1 members a detailed phylogram of putative TMKs of all three species *E. histolytica*, *E. dispar* and *E. invadens* was generated. The accession numbers of all TMKs used for this study are listed in Table S3. The kinase domain is present in only 28 EhTMKB1s, out of which only 13 members have complete kinase domain. The remaining members are classified as pseudokinase due to truncation or presence of stop codons and were not considered for the analysis (see Table S4) [8]. The phylogram clearly showed conservation of the orthologs among the three species as members of each family (A to F; as defined by Beck *et al.*) clustered separately, indicating functional conservation of the different classes across species (Figure 2) [12]. The TMKB1s of *E. histolytica* (EhTMKB1) and *E. dispar* (EdTMKB1) were leaves of common sub-branch, while *E. invadens* TMKB1 members (EiTMKB1) clustered in between the TMKB2 and TMKB3 members showing divergence from the other two species. This is not surprising as *E. invadens* sequences are known to be substantially different from that of other two *Entamoeba* species [18].

### Differential expression of EhTMKB1 members

**Figure 3:**
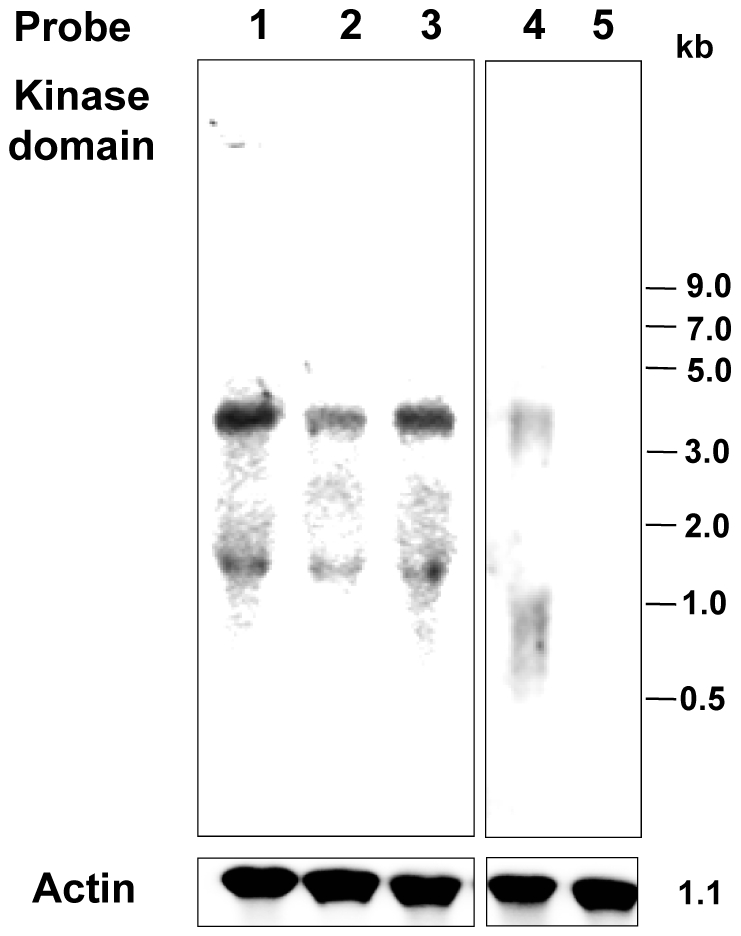
Expression of EhTMKB1 under different growth conditions. 30 µg of total RNA isolated from indicated cells was hybridized with a probe from the kinase domain of EhTMKB1. Lane 1, Normal proliferating *E. histolytica*; lane 2, serum starved *E. histolytica* (0.5% serum for 24 h); lane 3, serum replenished *E. histolytica* (0.5% serum for 24 h followed by 15% serum for 2 h); lane 4, proliferating *E. dispar*; lane 5, proliferating *E. invadens*. Actin gene was taken as loading control.

The presence of 35 members of EhTMKB1s raises the question regarding their biological function. Since many of these copies are truncated either at the extracellular domain or at the intracellular kinase domain, it is likely that some of these may be functionally divergent or inactive. It is also possible that different copies are expressed under different conditions. Thus, the expression analysis of EhTMKB1s was carried out to identify the member/s expressed in exponential growth and under stress conditions. Exponentially growing *E. histolytica* cells were subjected to serum starvation (0.5% serum for 24 h) followed by serum replenishment (15% serum for 2 h) and RNA was isolated at each stage and northern blots were hybridized with a probe derived from the kinase domain. The results are shown in Figure 3. Two bands of sizes 4.0 and 1.4 kb were obtained in the northern blots of RNA from exponentially growing cells (Figure 3, lane 1). Intensity of both bands was reduced to 42% in serum starved cells and on serum replenishment intensity increased to 75% within 2 h (Figure 3, lane 3). The 4.0 kb band was also seen in *E. dispar* (Figure 3, lane 4), but the *E. histolytica* probe did not hybridize with any band in *E. invadens* (Figure 3, lane 5). This may be due to low level of sequence identity (about 60% identity) at the nucleotide level between *E. histolytica* and *E. invadens* TMKB1s, confirming the separate branching of EiTMKB1 from the TMKB1s of *E. histolytica* and *E. dispar* (Figure 2).

### Identification of the expressed EhTMKB1 members

The expressed transcripts of EhTMKB1 family cannot be identified by northern analysis due to high level of sequence identity (≥95%) amongst all copies. Identification was done by sequencing the expressed copies, obtained after cloning the RT-PCR product. PCR primers were designed from a conserved region to amplify 400 bp fragment of the kinase domain. PCR conditions (input cDNA, no. of cycles) favored the identification of less abundant copies. For each sample (normal, serum starved and serum replenished) a minimum of 100 randomly picked clones were analysed in an experiment by nucleotide sequencing, and three independent experiments were conducted. The identity of the expressed copy was revealed by 100% match with the corresponding genomic copy. A representative alignment of clones obtained in different conditions is shown in the supplementary Figure S3.

**Figure 4:**
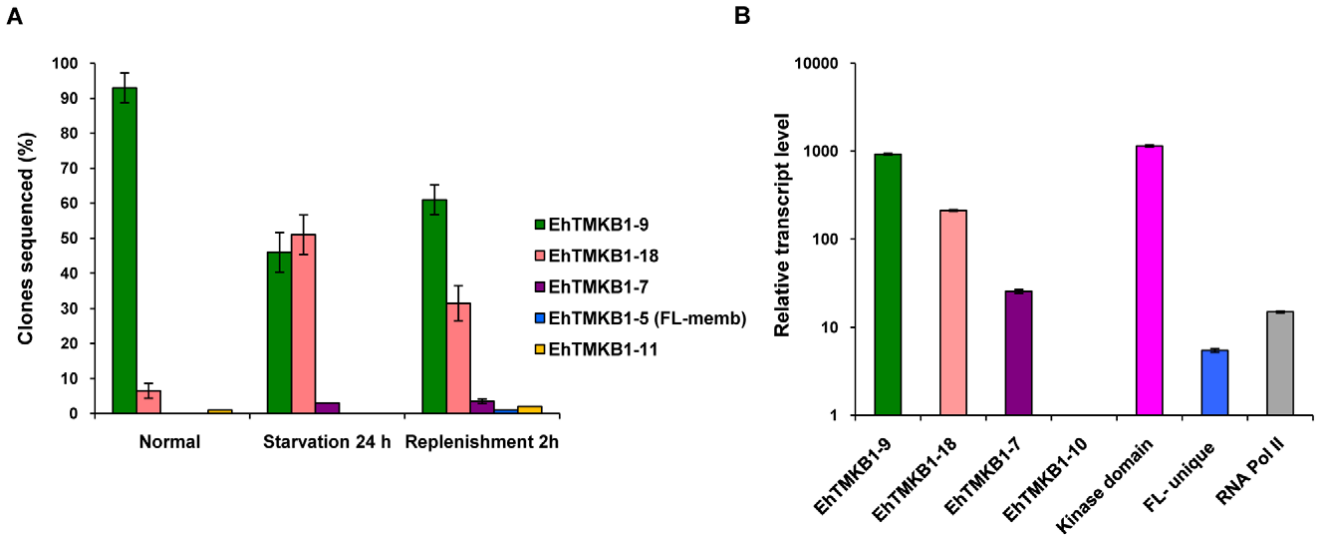
Identification and relative quantification of expressed EhTMKB1 members. (A) cDNA libraries were prepared from the kinase region of EhTMKB1 using RNA isolated from *E. histolytica* cells grown in different conditions as indicated. A hundred randomly chosen clones from each library were sequenced (repeated thrice). The sequences were mapped to specific EhTMKB1 members by sequence alignment. (B) Relative gene expression in normal proliferating *E. histolytica* cells using quantitative real-time PCR. Relative expression of EhTMKB1- 7, 9, 10, 18, EhTMKB1-kinase domain (kinase domain), EhTMKB1-full length unique region (FL-unique) and RNA polymerase II (RNA Pol II) was performed by qRT-PCR as described in “Materials and Methods”. The change in Ct values was calculated with reference to EhTMKB1-10 (least expressed EhTMKB1 member) and the fold difference in relative transcript level was plotted on logarithmic scale. All samples were analyzed in triplicates, in three independent experiments.

Results of sequence analysis showed that in exponentially growing cells 95% of the clones matched with the sequence of EhTMKB1-9 (Figure 4A). Hence, it is likely to be the predominantly expressed copy in exponential growing cells. In contrast, only 47% of the transcripts of serum starved cells matched with EhTMKB1-9 while 50% of the sequences in these cells were identified as EhTMKB1-18. On serum replenishment the fraction of EhTMKB1-9 sequences increased up to 61% while of EhTMKB1-18 was 32% (Figure 4A). Serum starvation down regulated the expression of EhTMKB1-9 while simultaneously up regulating the expression of EhTMKB1-18 from 4% in growing cells to about 50% in starved cells. Other EhTMKB1 transcripts identified by sequencing under different conditions are listed in the Figure 4A. To confirm that the results obtained were not due to selective amplification of certain cDNA sequences, the same PCR primers were used to amplify genomic DNA, and randomly picked clones were sequenced. No single homolog of EhTMKB1 was over represented in the sequences obtained, suggesting that there was no PCR-related bias for a particular copy (data not shown).

To quantitate the differential expression pattern, transcript level of a few selected EhTMKB1s - 7, 9, 10 and 18 were measured by quantitative real time PCR (qRT-PCR). Additionally, primers were also designed to amplify EhTMKB1- kinase domain (kinase domain; to study the total expression of all EhTMKB1s), EhTMKB1- full length unique region (FL-unique; to study the expression pattern of all full length members) and RNA polymerase II (unrelated control). The transcript levels were measured under normal proliferating condition. The list of real time PCR primers is given in Table 1. The primers for EhTMKB1- 7, 9, 10 and 18 were specific as these were designed from the N-terminal region of these genes which is not common with conserved part of EhTMKB1. We could not design specific primer pairs for other members due to ≥95% sequence identity at the nucleotide level. Therefore, we restricted our study to a few members. The primer pair for FL-unique was designed from the 5′- end which is conserved in all full length copies and not present in others (see Figure S1, blue colored unique region). The kinase domain primer pair is likely to amplify the transcripts from majority of EhTMKB1 members. The efficiency for each primer pair was calculated with serial dilution of genomic DNA and it lies within 1.96±0.06 which allowed us to directly compare the relative expression between the members [19].

**Table 1:** Real time PCR primers.

Gene product	Primer sequence (5′-3′)	
	Forward	Reverse
EhTMKB1-7	GTCAGACTCGAAAAACGCAAT	ATAGATAAACTACCCTCGAAAGTCATAC
EhTMKB1-9	TGACATATTATTTTTTATTGCTGACTTTA	TTGCATTATTGTCTGTTTCTTTTTG
EhTMKB1-10	TCTTTTTCATTTCATTTTGTATTTCTG	GGTACTGTATTTGAATTGATTTTTTCA
EhTMKB1-18	TGTTTTTGATGGTTTTGTTTCAATA	TCAATGTAGCAAAAGTATAGTGTTGAG
FL-unique	TGTATTCATGCTAAAGCTTATGATTG	CATTCTTAGTTGTTTTGCATAGTGTAA
Kinase domain	ATCAAATTTATGATAGATGGAGCAA	ATTATCTGGTTTAATATCTCGATGTAATATT
RNA Pol II	GAGTCATGTTCTTCAAGGTTTTTTACTTT	CTTCGATACCTCCATTTAAGTGTTCA

Annealing temperature - 58°C.

Results obtained by qRT-PCR showed that EhTMKB1-9 is the predominantly expressed EhTMKB1 member (Figure 4B). The EhTMKB1-9 transcript levels were 5 and 180 fold more than that of the EhTMKB1-18 and full length members respectively under proliferating condition (Figure 4B).

### Expression of EhTMKB1-18 under serum starvation

**Figure 5:**
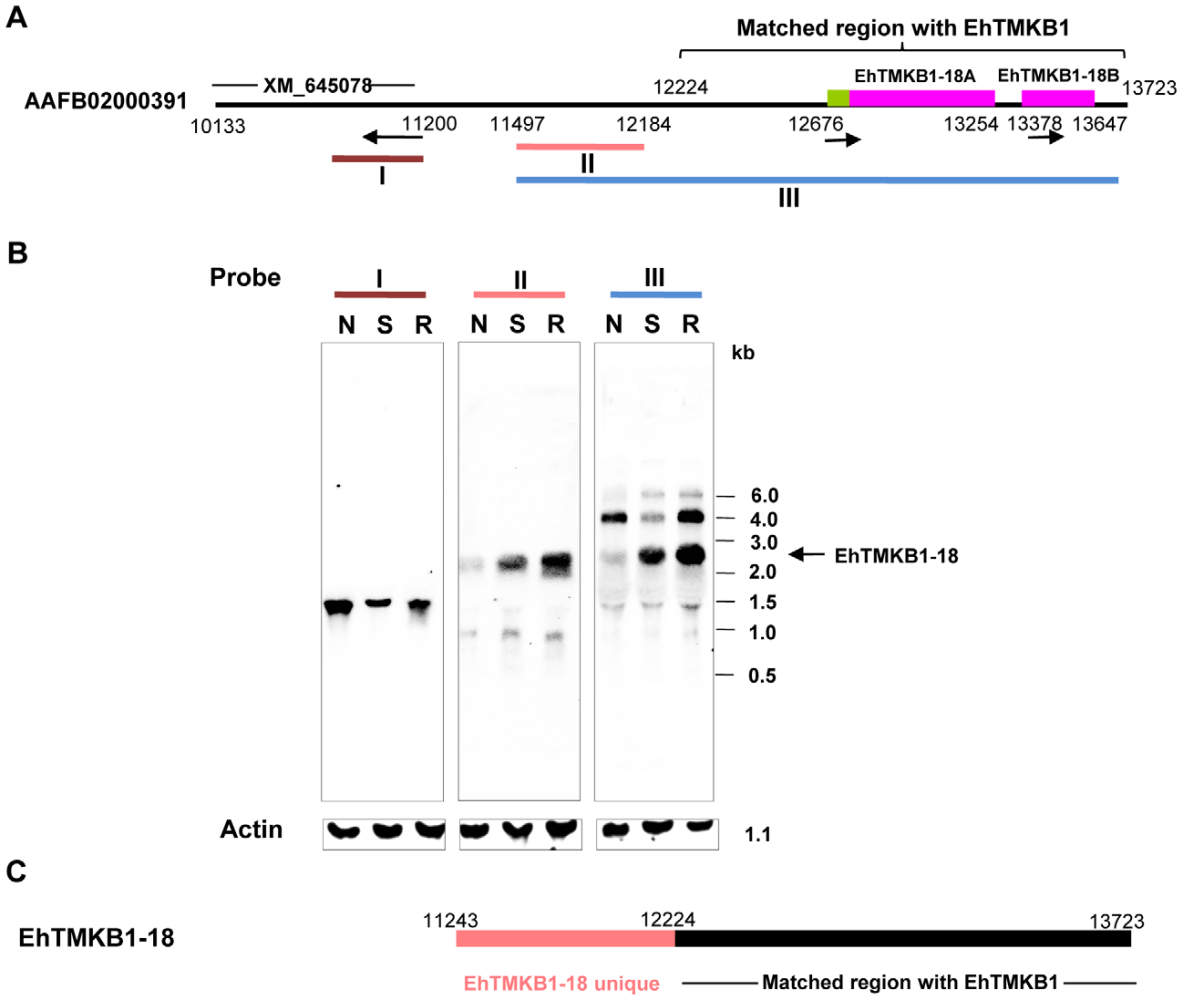
Induction of EhTMKB1-18 expression on serum starvation. (A) Schematic representation of contig AAFB02000391. EhTMKB1 matched region and annotated genes (XM_645078, EhTMKB1-18A and 18B) are marked. The arrows represent the direction of transcription of the putative annotated genes. (B) Northern hybridization using probes derived from regions marked as I, II and III was performed with RNA isolated from cells treated under different conditions. Normal (N), serum starvation (S) and replenishment conditions (R). Actin gene was used as loading control. (C) Schematic representation of EhTMKB1-18.

From previous data (Figure 4A) it was shown that the expression of EhTMKB1-18 is stimulated after serum starvation. This sequence is located in the contig AAFB02000391 and the two genes (EhTMKB1-18A of 579 bp and EhTMKB1-18B of 261 bp) have been annotated to map in this sequence (Figure 5A). However the 400 bp RT-PCR amplicon sequenced in Figure 4A encompassed both the genes (EhTMKB1-18A and 18B). Therefore, either the two genes are co-transcribed or there is an error in the annotation. The latter seems to be the case as the RT-PCR followed by southern analysis with probe specific for contig AAFB02000391 showed that the transcript of EhTMKB1-18 gene is much bigger in size as compared to the expected size for annotated genes EhTMKB1-18A and 18B (data not shown). In order to determine the transcript length of EhTMKB1-18, northern blot analysis was performed with probes from three different regions of contig AAFB02000391 as shown in Figure 5A.

Probe III comprising of 5′- sequence upstream of the annotated genes EhTMKB1-18A and 18B and 3′- conserved sequence shared by other EhTMKB1 members, hybridized to essentially three RNA species, 4.0 kb, 2.6 kb and a minor band at 1.4 kb (Figure 5B, Probe III). The 4.0 and 1.4 kb bands were also seen in Figure 3 and may be due to the conserved kinase domain present in probe III. The 2.6 kb band is likely to be the transcript of EhTMKB1-18, as the expression was induced on serum starvation. This was confirmed by using upstream probe II that is specific for EhTMKB1-18 and is upstream of the two annotated genes. This probe hybridized with a 2.6 kb band whose intensity increased many fold on serum starvation suggesting that EhTMKB1-18 is indeed a serum starvation induced gene. The results also suggest that the two annotated genes EhTMKB1-18A and 18B are actually part of one transcript of EhTMKB1-18. To confirm that EhTMKB1-18 does not encompass another annotated gene XM_645078 present upstream, probe I from this gene was used in northern blot analysis. It hybridized to a band of about 1.3 kb. The intensity of this band was reduced on serum starvation indicating that it is a separate gene product (Figure 5B). The results clearly show that EhTMKB1-18 encodes a serum starvation induced transcript of 2.6 kb with N-terminal ∼0.9 kb region unique to this member and the remaining 1.7 kb similar to other EhTMKB1s. Based on the results presented here the annotation of the contig AAFB0200391 with respect to EhTMKB1 is shown in Figure 5C.

### Expression of EhTMKB1-9

**Figure 6:**
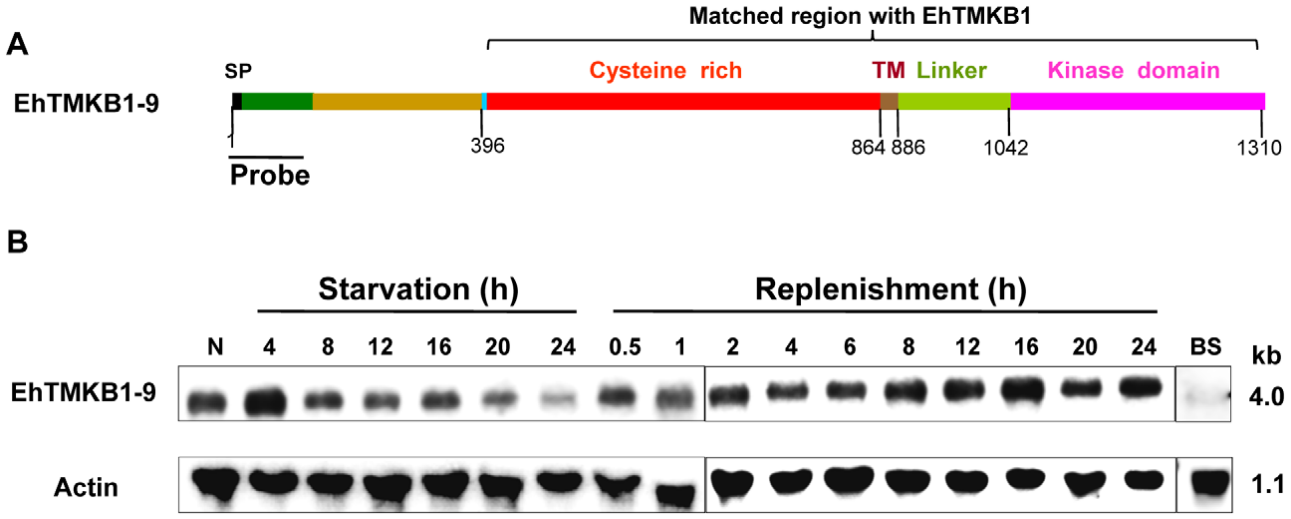
Domain structure and expression analysis of EhTMKB1-9. (A) Schematic representation of different regions of EhTMKB1-9 based on amino acid composition is indicated schematically. N-terminal region (470 bp- green color) is unique to this gene and is not present in any other EhTMKB1 member. This part was used as a specific probe in rest of the studies. Signal peptide sequence (SP), trans-membrane region (TM). (B) Northern hybridization with EhTMKB1-9 specific probe was performed with RNA isolated from cells undergoing different treatments and at time points as indicated. N denotes normal proliferating *E. histolytica* cells. BS indicates replenishment of the starved cells with boiled serum (100°C for 5 min) for 2 h. Actin gene was used as loading control.

Differential expression of EhTMKB1-9 was demonstrated by northern analysis using a specific probe. This probe (470 bp) was selected from the 5′-end of the ORF of EhTMKB1-9 that did not show match with any other *E. histolytica* sequence, including full length copies of EhTMKB1 (Figure 6A and S1). As expected, the probe hybridized to a band of about 4.0 kb from total RNA of normal proliferating cells. The band intensity decreased to 24% after 24 h of serum starvation. On serum replenishment the amount of transcript increased back to normal level within 2 h (Figure 6B). Nearly 1.7 fold increase in the transcript level was observed after 16 h of serum replenishment and there after the level of EhTMKB1-9 normalized. The factor involved in serum-induced expression of EhTMKB1-9 gene product appears to be heat labile as the replenishment of serum starved cells with boiled serum did not induce the EhTMKB1-9 transcript levels (Figure 6B).

### Expression analysis of EhTMKB1-9 and 18 by qRT-PCR

**Figure 7:**
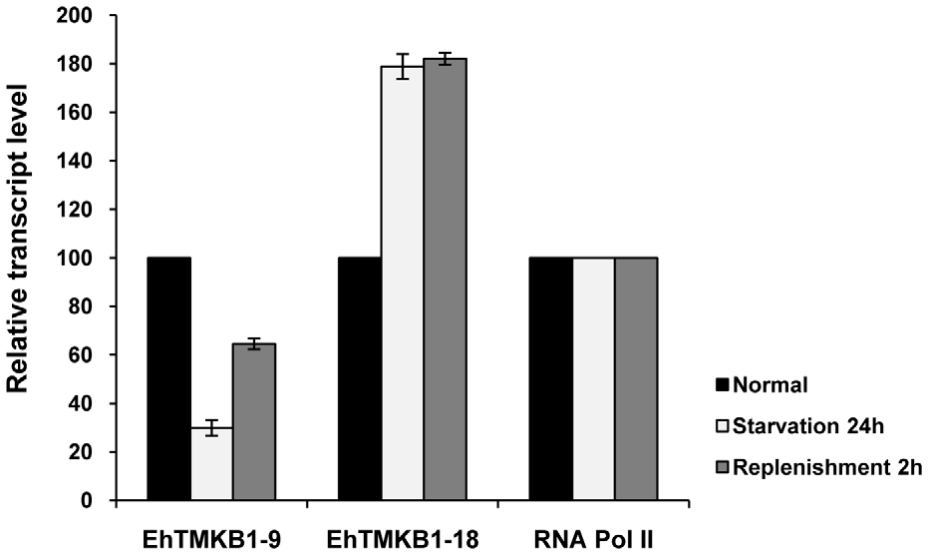
Expression analysis of EhTMKB1-9 and 18 by qRT-PCR. Quantitative real-time PCR was performed on EhTMKB1-9, 18 and the RNA polymerase II gene in normal, starvation and replenishment conditions. All samples were analyzed in triplicates, in three independent experiments. Values are normalized to the endogenous control (RNA Pol II) and results are expressed as fold change in percentage to the normal condition (taken as 100%).

In order to quantify the change in transcript level of EhTMKB1-9 and 18 under different conditions, real-time PCR was performed. To allow comparison between time points, results were normalized to the values obtained for RNA polymerase II gene [12]. The fold difference in transcript levels was calculated keeping expression in normal conditions as 100%. The level of EhTMKB1-9 transcript was reduced to 30% while that of EhTMKB1-18 increased by 80% on serum starvation (Figure 7). The serum replenishment for 2 h resulted in an increase of EhTMKB1-9 transcript to 65% of the levels in exponential growth while the EhTMKB1-18 transcript level remained unchanged during this duration. The results are in agreement with the library sequencing and northern analysis data (Figure 4, 5B and 6B).

### Mapping 5′ and 3′- ends of EhTMKB1-9

**Figure 8:**
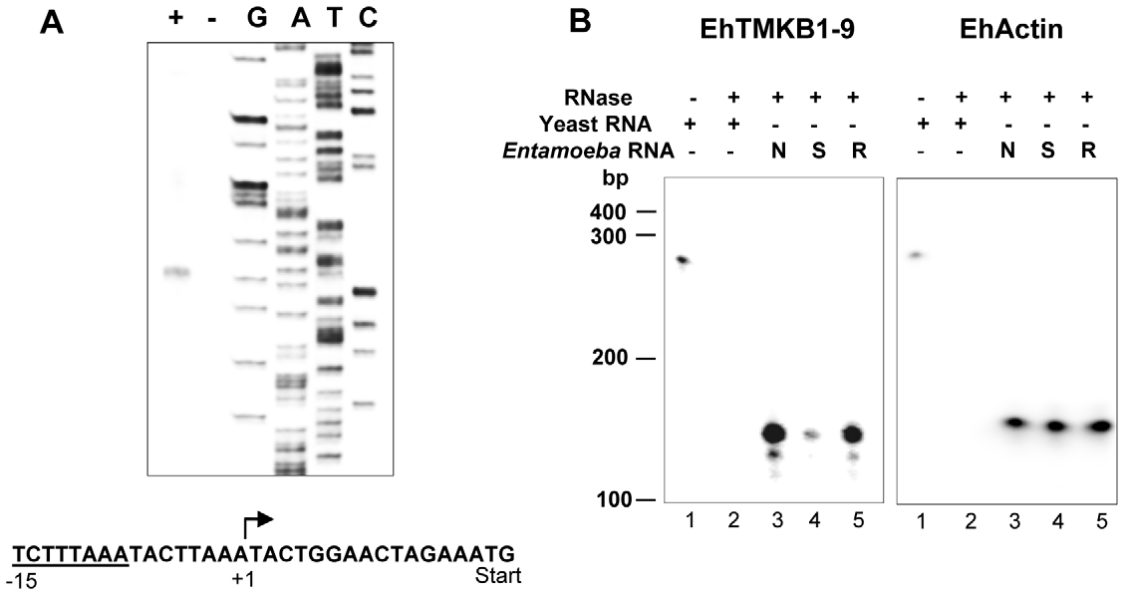
Mapping the transcription start point (TSP) of EhTMKB1-9. (A) Primer extension products were analyzed by electrophoresis and the transcription start site was identified using a sequencing reaction separated alongside (see “Materials and Methods”) and marked in the displayed sequence (+1) along with ATG as start codon and putative TATA box (underlined). (B) The RNase protection assays with 30 µg of RNA isolated from proliferating (N, lane 3), serum starved for 24 h (S, lane 4), serum replenished for 2 h (R, lane 5) or yeast (lanes 1, 2) cells using a probe derived from −111 to +147 of EhTMKB1-9 gene or with a probe derived from EhActin gene (loading control) was performed as described in “Materials and Methods”.

The transcription start point (TSP) of EhTMKB1-9 was mapped by primer extension. An antisense oligonucleotide, spanning from 107 to 131 nucleotides downstream of the putative start codon, was used. A major extension product of 147 nucleotides was obtained (Figure 8A). Thus, the TSP was mapped 15 bases upstream of the translation start codon. A search for the consensus sequence described for *E. histolytica* core promoter and upstream regulatory elements revealed the presence of 8 bp TATA box-like sequence TCTTTAAA, located 15 bp upstream to the transcription initiation site [20]. The analysis also showed the presence of previously reported conserved elements (CE1b) TAAATCAA and (CE2) GAAC, upstream to the transcription start point [21].

RNase protection assay was performed to confirm the TSP and to quantitate the transcript levels of EhTMKB1-9 under different conditions. Labeled antisense RNA was generated from the region −111 to +147 by *in vitro* transcription, and was used in the protection assay as described in “Materials and Methods”. The size of the protected fragment was 147 nucleotides (run on 6% urea PAGE along with a sequencing reaction - data not shown) confirming the TSP mapped by primer extension (Figure 8B). The relative intensities of the protected bands provided the quantitative estimate of changes in the transcript levels. When compared to normal cells, the amount of EhTMKB1-9 transcripts decreased to 14% after 24 h serum starvation while after 2 h of serum replenishment the value was 73%.

The 3′- end was determined by cloning the PCR product from 3′- RACE (Rapid amplification of cDNA ends) and around 30 randomly picked colonies were analyzed by sequencing. The 3′- untranslated region (UTR) of EhTMKB1-9 was deduced to be 17 bp in length with the sequence TGATTGAAACTATTGATATT -(A)_n_ where TGA is the stop codon. Some of the known amoebic signals, such as putative polyadenylation signal sequence TATTT and a stretch of pyrimidine residues were also present adjacent to poly(A) tail [20]–[22]. Both 5′- and 3′- UTRs of this gene were less than 20 nucleotides, similar to the UTRs of most *E. histolytica* genes.

### Serum response is mediated by upstream sequences of EhTMKB1-9

**Figure 9:**
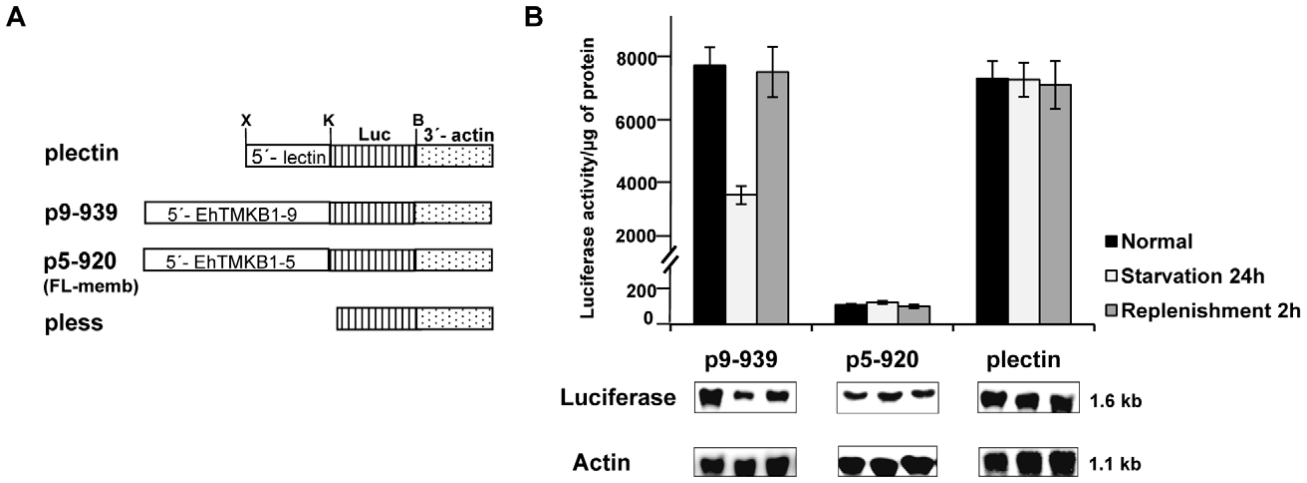
Promoter analysis of EhTMKB1-9 using luciferase as a reporter gene. (A) Schematic representation of the promoter constructs used in the study. X, K, B are *Xho*I, *Kpn*I and *BamH*I sites respectively and Luc represents luciferase gene. (B) Luciferase expression was determined by measuring the reporter activity and by northern hybridization using luciferase gene probe. For these studies stable transfectants of the indicated constructs were used and each cell line was subjected to three different conditions - normal proliferation, serum starvation and replenishment. Actin gene was used as loading control.

The promoter activity of EhTMKB1-9 gene was demonstrated by cloning the upstream fragment (−939 to +15) next to a luciferase reporter gene using the vector pEh-Neo-luc-S (plectin) as described in “Materials and Methods”. For comparison, a luciferase construct was also prepared using 920 nucleotides upstream of the start codon from a full length member EhTMKB1member (EhTMKB1-5; p5–920) in the same vector. As a control, a promoter-less vector (pless) was constructed in which the complete promoter region of the parent vector was removed as shown in Figure 9A. These plasmids were electroporated in trophozoites and stable transfectants were selected using the drug G-418.The luciferase activity of different transfectants is shown in Figure 9B. The transfectants containing the upstream sequences of EhTMKB1-9 (p9–939) displayed very high level of luciferase activity (about 8000 fold higher than that of the pless) whereas those with the upstream sequences of EhTMKB1-5 (p5–920) showed only 150 fold higher activity compared with pless. The luciferase activity of transfectants was also checked in cells subjected to serum starvation. The levels of luciferase in plectin and p5–920 containing cells remained unchanged upon serum starvation and replenishment. However, the luciferase expression from EhTMKB1-9 promoter decreased to 50% after 24 h of serum starvation, and reverted back to 88% within 2 h of serum replenishment (Figure 9B). Transcript levels in these transfectants were also directly measured by northern hybridization of total RNA with luciferase probe (Figure 9B). The results clearly show that the promoter of EhTMKB1-9 is responsive to serum.

**Figure 10:**
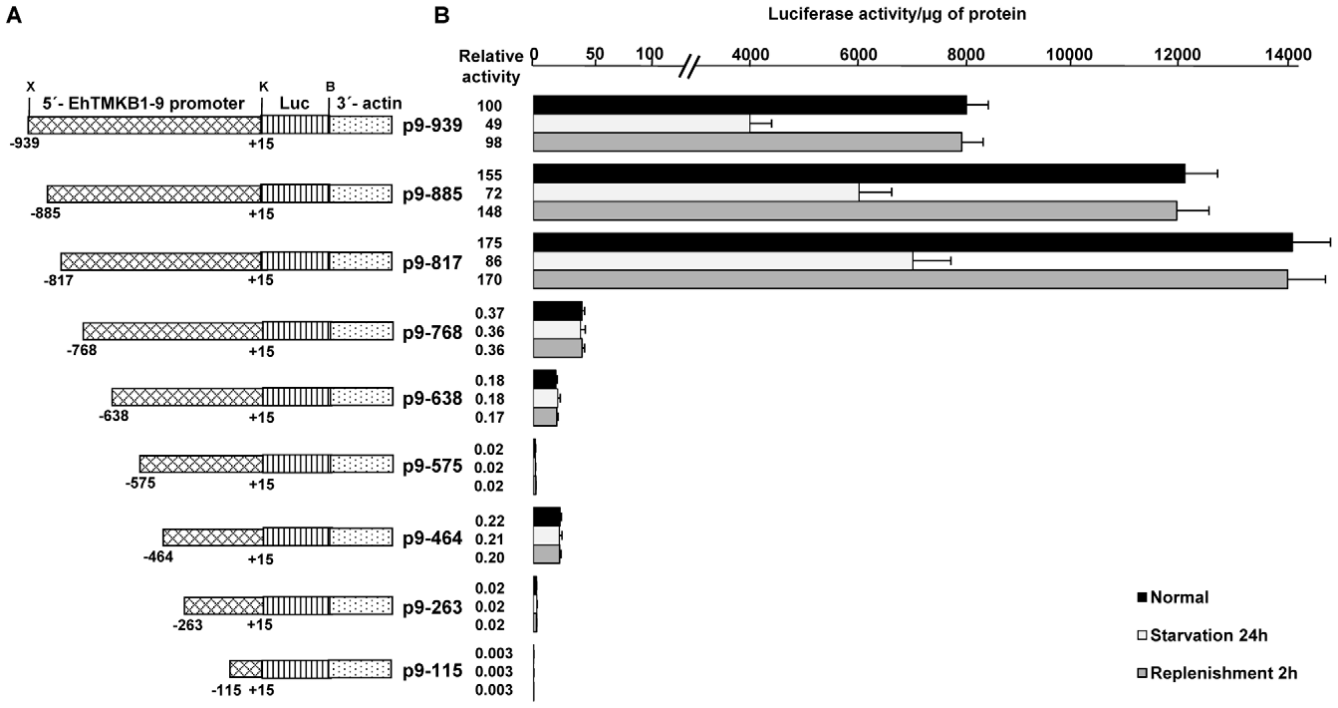
Mapping the upstream region of EhTMKB1-9 required for serum response. (A) Schematic representation of EhTMKB1-9 promoter deletion constructs containing upstream sequences with defined positions cloned upstream to reporter luciferase (Luc) gene. X, K, B are *Xho*I, *Kpn*I and *BamH*I sites respectively. (B) Reporter luciferase activity of the stable transfectants under different conditions - normal proliferation, serum starvation and replenishment. Activity of p9–939 under normal conditions is taken as 100 for relative fold calculation.

To determine the minimum sequences required for promoter activity and serum responsiveness, deletions were made from 939 bp upstream fragment of EhTMKB1-9 ORF (Figure 10A). Stable transfectants were generated and reporter luciferase assays were performed. The deletion construct comprising region −768 to +15 (p9–768) showed 260 fold reduction in luciferase activity compared to p9–939 (Figure 10B). Detectable activity was seen in the construct containing the region −464 to +15 (p9–464) while further deletions gave negligible activity. The luciferase activity showed that the region −939 to −768 is required for promoter activity and is responsible for the serum responsiveness of EhTMKB1-9. Interestingly, deletion of the region between −939 to −817 resulted in 1.75 fold increase in luciferase activity as compared to p9–939, indicating that this region could act as a negative repressor of EhTMKB1-9 expression (Figure 10B).

### EhTMKB1-9 is a protein kinase

**Figure 11:**
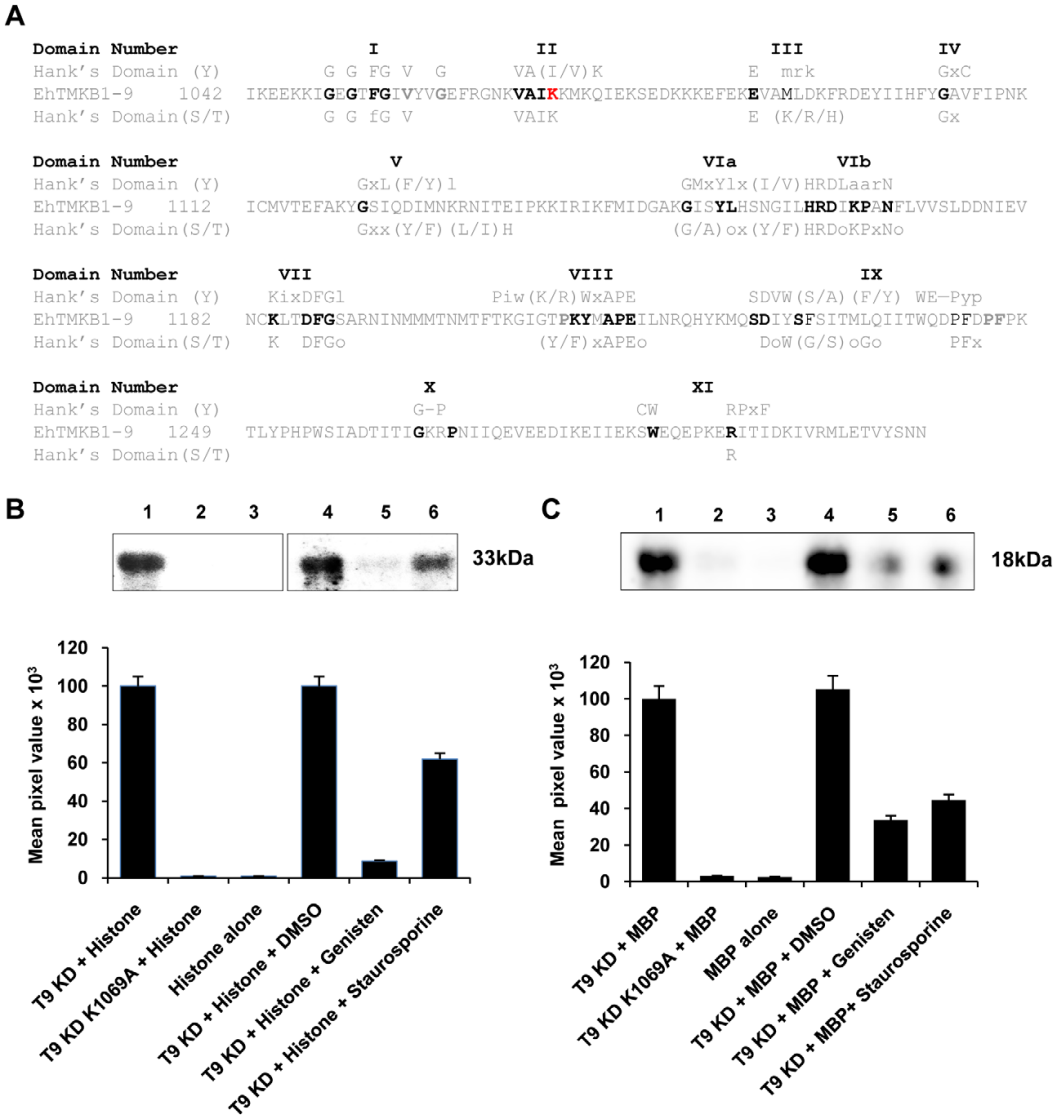
Protein kinase activity of EhTMKB1-9. (A) Alignment of EhTMKB1-9 kinase domain with Hank's consensus conserved residues for serine/threonine or tyrosine kinase. Upper cased residues are conserved, and non conserved positions are denoted by “x,” whereas positions requiring hydrophobic residues are denoted by “o”. (B) and (C) *In vitro* phosphorylation assay was performed with 0.5 µg purified EhTMKB1-9 kinase domain (T9KD) or with the K1069A (site directed mutant of T9KD) using histone type IIIS or myelin basic protein (MBP) as substrates. The protein kinase inhibitors genisten (10 µg/ml) or staurosporine (50 nM) were used as indicated. The reaction was initiated by adding [γ-^32^P] ATP and was terminated by adding Laemmli buffer and boiling for 5 min. The products were analysed on 12% (for histone) or 14% (for MBP) SDS-PAGE and scanned in a phosphorimager. The top panel shows the [^32^P]-labeled bands of histone (33 kDa) or MBP (18 kDa).

Bioinformatics analysis clearly showed that EhTMKB1-9 contains a functional protein kinase domain as all known conserved residues for protein kinases were present. The sequence contained the necessary residues for both serine/threonine and tyrosine kinase family members (Figure 11A) [23]. In order to show that the domain indeed has protein kinase activity, the intracellular kinase domain (amino acid positions 1041–1309, 32 kDa) was cloned, expressed and purified from *E. coli* as described in “Materials and Methods”. A putative kinase dead mutant (K 1069 A) was generated through site directed mutagenesis and the protein was purified. The kinase activity was tested using histone type III and myelin basic protein (MBP) as substrates (Figure 11B and 11C). Phosphorylation was observed with wild type protein (T9KD) but not with the kinase-dead mutant (T9KD K1069A). While genisten, a tyrosine kinase inhibitor [24] decreased the level of histone phosphorylation by about 90% and MBP phosphorylation by 70%, staurosporine, a serine/threonine kinase inhibitor [25] inhibited histone phosphorylation by 40% and that of MBP by 55%. These results show that the EhTMKB1-9 kinase domain may have both ser/thr and tyr kinase activities, which is similar to the dual specificity Sp1A protein kinase of *D. discoidium*, a homolog of TMKs [13].

### Cell surface localization of EhTMKB1-9

**Figure 12:**
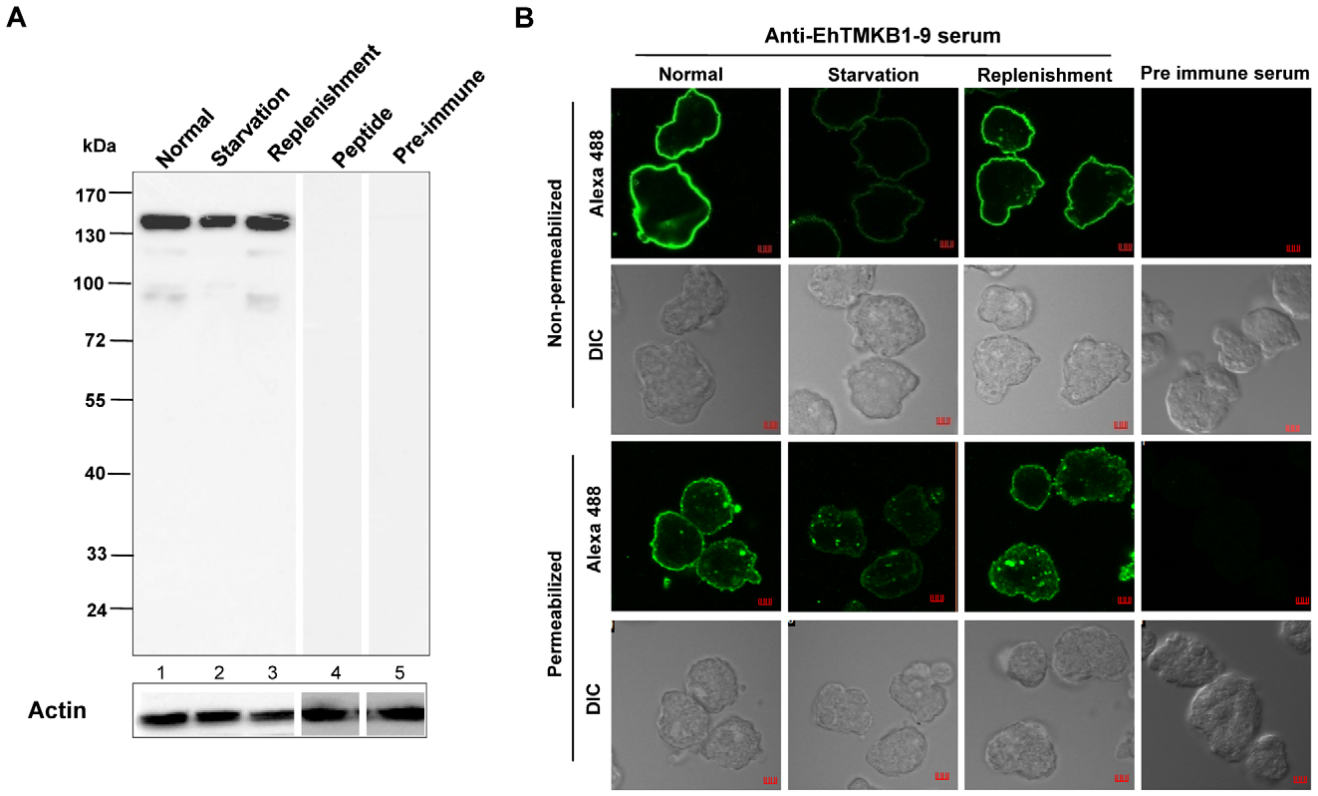
Expression and immunolocalization of EhTMKB1-9 in *E. histolytica*. (A) Western blot with affinity purified anti-EhTMKB1-9 peptide antibodies was performed using amebic lysates in different conditions - normal proliferating cells (lane 1), serum starvation (lane 2) and replenishment (lane 3), or in presence of 50 µM competing peptide (lane 4), or pre-immune serum (lane 5). The blots were stripped and re-probed with anti-EhActin antibody. (B) Confocal microscopy of non-permeabilized and permeabilized (0.1% Triton X-100) *E. histolytica* trophozoites stained with pre-immune serum or affinity purified anti-EhTMKB1-9 antibodies. The growth conditions are described in panel A. Bar represents 5 µm. Magnification 60×.

Rabbit antibodies were raised against a peptide specific for the extracellular domain of EhTMKB1-9 (LYDYSKYKSVIIRFS). It recognized a protein band of 140 kDa in normal proliferating cells on western blots (Figure 12A).This band disappeared when antibody was pre-absorbed against 50 µM of the antigenic peptide (Figure 12A, lane 4). Affinity purified EhTMKB1-9 antibodies stained the surface of non permeabilized as well as permeabilized trophozoites. The stain was uniformly distributed throughout the cell surface. Staining was minimal upon serum starvation and was regained on serum replenishment (Figure 12B). These results show that EhTMKB1-9 is localized to the *E. histolytica* trophozoite surface as type I integral membrane protein under normal growth conditions and is redistributed upon serum starvation and replenishment. The change in expression pattern of the EhTMKB1-9 is in agreement with its proposed function of sensing the changes in the external environment.

### Effect of down regulation of EhTMKB1-9 on cellular proliferation

**Figure 13:**
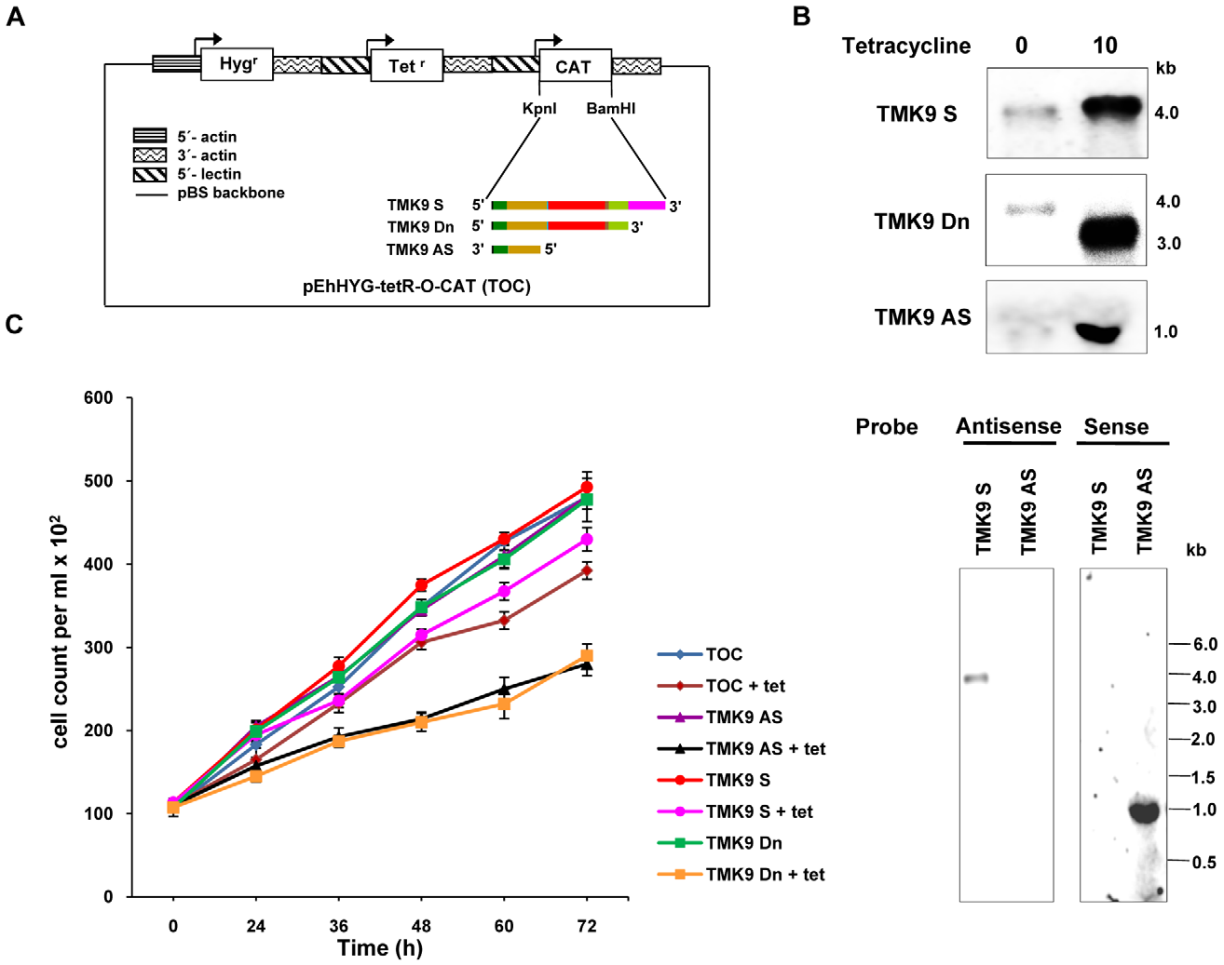
Down regulation of EhTMKB1-9 decreases cellular proliferation. (A) Schematic representation of pEhHYG-tetR-O-CAT vector (TOC), showing EhTMKB1-9 in sense (TMK9-S), dominant negative mutant lacking kinase domain (TMK9-Dn) or 1.0 kb region of the ORF (from N-terminal) of the EhTMKB1-9 gene in antisense orientation (TMK9-AS). (B) Northern hybridization with EhTMKB1-9 specific probe (double stranded) was performed with RNA isolated from transfectants grown for 48 h either in presence or absence of 10 µg/ml tetracycline in upper panel. The lower panel shows northern hybridization with strand specific EhTMKB1-9 probe with RNA isolated from TMK9-S and TMK9-AS grown for 48 h in presence of 10 µg/ml tetracycline. (C) Growth was measured in the absence or presence of 10 µg/ml tetracycline (added to the cultures at zero time).

The EhTMKB1-9 gene was cloned either in sense or antisense orientation in the tetracycline-inducible expression vector pEhHYG-tetR-O-CAT (TOC) as shown in Figure 13A [26]. To attain a dominant negative phenotype, a mutant EhTMKB1-9 without kinase domain was also cloned in TOC. The resulting transfectants, TMK9-S (full length EhTMKB1-9 in sense orientation), TMK9-Dn (mutant lacking kinase domain) and TMK9-AS (initial 1.0 kb of ORF in antisense orientation) were checked for RNA expression and cellular proliferation before and after induction with 10 µg/ml tetracycline. Induction with tetracycline showed a four and twenty fold increase in TMK9-S (4.0 kb) and TMK9-Dn (3.2 kb) transcripts respectively (Figure 13B upper panel). In TMK9-AS transfectants, a high level of antisense TMK9 transcripts was observed in the presence of tetracycline which was further confirmed by strand specific probe (Figure 13B lower panel). Both TMK9-Dn and TMK9-AS transfectants displayed slower multiplication as the cell numbers were reduced by 40% at 72 h (Figure 13C). Similar results were obtained in two independent transfected cell lines. The growth of TMK9-S cells was largely unaffected by tetracycline showing a slight reduction in cell number (10–15%) in the presence of tetracycline. The growth defect was reversible, as removal of tetracycline from the culture restored normal growth. Moreover, these slow growing cells were viable and did not take up trypan blue. The decrease in cellular proliferation on over expression of TMK9-Dn and TMK9-AS clearly suggests that EhTMKB1-9 plays an essential role in the growth of *E. histolytica* trophozoites.

### Adhesion and target cell killing by EhTMKB1-9 transfectants

**Figure 14:**
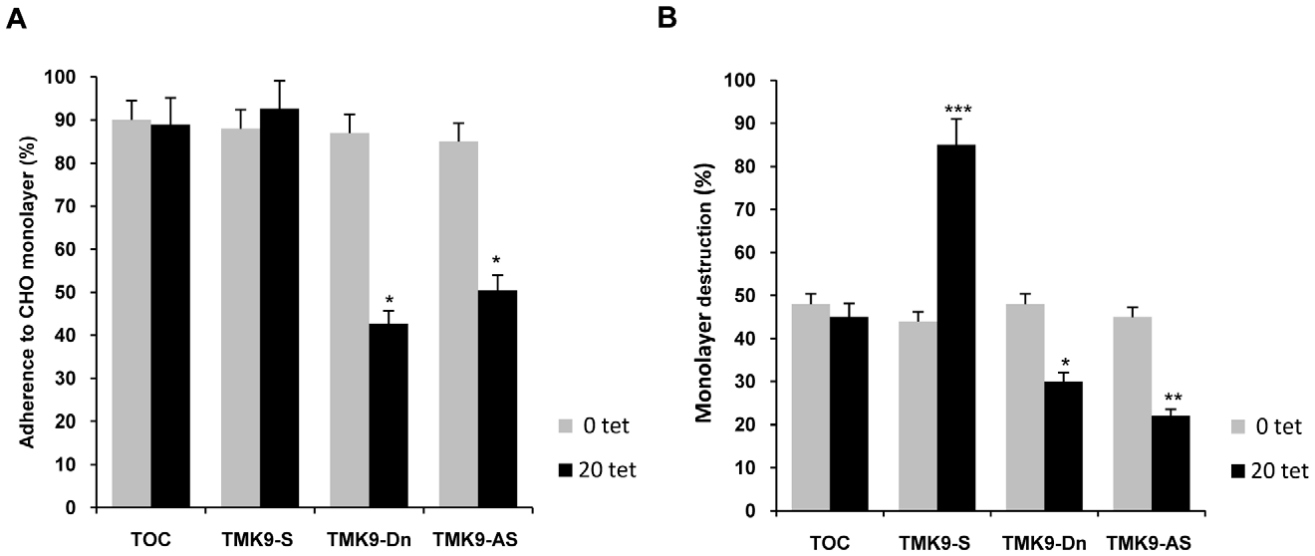
Adhesion and target cell killing by EhTMKB1-9 transfectants. (A) Adhesion of EhTMKB1-9 transfectants (2×10^5^) to fixed monolayers of CHO cells after 30 min of interaction. The trophozoites that remained attached to the monolayer after washing were counted. Comparisons were made with TOC vector control grown at 20 µg/ml of tetracycline and the statistical significance was determined by paired- *t* test. *, p<0.01. (B) The destruction of CHO monolayer by EhTMKB1-9 transfectants. The killing of CHO cells was determined by methylene blue staining. Indicated amebic cells were used at a ratio of 1∶1 with respect to CHO cells. The cells were grown at 20 µg/ml hygromycin B and in the absence or presence of 20 µg/ml tetracycline for 48 h. TOC, original tetracycline-inducible vector containing cell line; TMK9-S and TMK9-AS are cells containing EhTMKB1-9 gene in sense and antisense orientations respectively and TMK9-Dn is dominant negative mutant (kinase domain deleted) of EhTMKB1-9. Comparisons were made with TOC vector control grown at 20 µg/ml of tetracycline and the statistical significance was determined by paired-*t* test. *, *p*<0.01; **, *p*<0.005; ***, *p*<0.002.

The ability of the human intestinal parasite *E. histolytica* to destroy its target cells can be separated into two steps: recognition and adhesion to target cells followed by killing and phagocytosis [27]. In order to test whether EhTMKB1-9 is involved in these processes, adhesion and cytopathic assays were performed with EhTMKB1-9 transfectants and CHO cells as target. No change in adhesion level was observed when tetracycline was not added to the culture (Figure 14A). The adhesion levels of full length TMK9-S were equivalent to that of vector control (TOC). However, the transfectants over-expressing TMK9-Dn and antisense RNA (TMK9-AS) showed a significant (45%) decrease in adhesion to fixed CHO cells (Figure 14A).

In contrast to adhesion, the transfected trophozoites expressing EhTMKB1-9 gene in sense orientation showed a 40% increase in their ability to destroy CHO monolayer over that of TOC cells (Figure 14B). However, a significant (18%) decrease in cytopathic activity was observed when TMK9-Dn and TMK9-AS cells were used for target cell killing suggesting the involvement of EhTMKB1-9 in parasite virulence (Figure 14B).

## Discussion

The *E. histolytica* genome encodes an array of novel molecules such as calcium binding proteins and TMKs that are likely to be involved in different signal transduction processes [11], [28]. Ninety TMKs were identified on the basis of sequence similarity and consensus motifs. *E. histolytica* may require all of these molecules to sense its external environment (for example, changing environment in the gut) and respond appropriately. In other organisms TMKs are known to play a critical role in perception and transduction of these external signals. It is therefore, reasonable to speculate that amebic TMKs may also have similar functions. Many of the mammalian TMKs have been worked out in great detail in terms of identification of extracellular ligands and intracellular kinase substrates [29]–[31]. Plants also encode a large number of TMKs and functional assignment of a few of these has been reported [3], [4], [32]. However, none of the *E. histolytica* TMKs has been characterized in detail. The putative functions of two such members, EhTMKB1-2 (B1.I.1, a EhTMKB1 full length member; Figure S1B) in cellular proliferation and EhTMKB3-96 (PATMK96) in phagocytosis have been suggested [14], [15]. In this report, we present evidence to show that EhTMKB1-9 is a serum induced TMK and we suggest that it may have an important role in cellular proliferation and virulence.

From nucleotide sequence analysis we identified 35 members of EhTMKB1 family, most of which share the highly conserved C-terminal kinase domain. Putative full length sequences were found only in six members. It seems that the N-terminal ends are much more divergent as compared to other regions. Since different copies may have arisen due to duplication and divergence, it is possible that part of a gene copy, particularly 5′- and 3′- ends may have been acquired from surrounding sequences following the insertion of a duplicated copy. This appears to be the case for EhTMKB1-9 where 5′- end of the gene does not have similarity with other copies. It was possible for us to validate/modify some of the predictions from bioinformatics analysis as we could design specific probes for some of the members and detected specific transcripts (for example EhTMKB1-7, 9, 10 and 18). Where specific probes could not be defined it was difficult to validate annotation of gene structure. It is also clear from our phylogenetic analysis that EhTMKB1 was present before the split of *E. dispar* and *E. histolytica*. However, the expansion of the family may have taken place after the divergence of the two species as these clustered separately (Figure 2). Since the sequence identity among the members is very high (≥95%) it is likely that the expansion of EhTMKB1s is a recent event. It is difficult to comment on *E. invadens* TMKB1s as sequence divergence is high and these are placed ambiguously in the phylogram.

Our data clearly shows that there is a high degree of selectivity in the expression of different members of EhTMKB1 family and that the expression of a specific copy is probably determined by external condition. The results show that expression of EhTMKB1-9 is serum responsive and transcriptionally regulated. The promoter elements of only a few *E. histolytica* genes have been characterized so far and these elements have generally been found close to the transcription start sites [21], [33]–[35]. However, a positive regulatory element of EhTMKB1-9 was mapped from −817 to −768 and a negative regulatory element (−939 to −817) was found in close proximity to it. Presence of both positive and negative regulatory sites has also been observed for other *E. histolytica* promoters [33], [36]–[38]. It appears from our experiments that the basal promoter elements may not also reside close to the transcription start site but probably around −768 to −638 nucleotides upstream. We need to carry out further mapping to clearly pinpoint the site. This is one of the rare examples of an inducible promoter in a protozoan parasite. Availability of such a system will help to decipher signal transduction and inducible transcription mechanisms in *E. histolytica*.

EhTMKB1-18 is part of a growing list of genes whose expression is induced by serum starvation. Jain *et al.*, have observed stimulation of expression of a dynamin-like-gene on serum starvation [39]. Therefore, it is likely that *E. histolytica* may have a system for responding to serum starvation by stimulating the expression of a number of genes whose products may be necessary for survival. Nitrogen and carbon starvation have been studied extensively in many other systems but not much in protozoan parasites [40]–[44]. The role of EhTMKB1-18 in serum starvation is not clear as the sequence is unlikely to encode any protein product and it has not been annotated as a gene. The possibility exists that the transcript could be a non coding RNA with regulatory role. It is likely that EhTMKB1-18 transcript may help cells to cope with the effect of serum starvation as RNA levels stay high even after serum replenishment until exponential growth is achieved.

In our earlier report, EhTMKB1-2 (B1.I.1, Figure S1) a full length member was shown by us to be expressed in *E. histolytica* cells and involved in serum-induced cellular proliferation [14]. The expression of full length copies was found to be at a much lower level (180 fold less, Figure 4B) as compared to that of EhTMKB1-9. In addition, their expression was not modulated by serum (p5–920; a full length member, Figure 9B). Moreover, the full length members were localized more to the frontal and posterior regions of the trophozoite while EhTMKB1-9 was uniformly distributed. It is possible that different EhTMKB1 full length members may have different roles. At present it is difficult to distinguish among these members as none of the reagents (antibodies, gene probes and primers) are specific for a particular full length copy. It appears from our previous and current studies that the main function of EhTMKB1s is cellular proliferation and serum response. However, the individual copies may have specific functions pertaining to the same theme, which may allow a large diversity of responses.

Our data show that the protein encoded by EhTMKB1-9 has functional kinase activity. Interestingly, the kinase domain of EhTMKB1-9 has features of both ser/thr and tyr kinases similar to other dual specificity kinases [45]. Inhibition of kinase activity in presence of ser/thr and tyr kinase inhibitors confirmed the dual specificity of EhTMKB1-9. However, the kinase activity was visualized using external substrates and no significant autophosphorylation was observed in any of the experiments (data not shown). This suggests that unlike other TMKs this protein may not have autophosphorylation activity or it was undetected due to the following possible reasons: (a) requirement of di/oligo-merization of TMK molecules [1], [26], [30]; (b) the kinase domain alone may not be sufficient and the complete molecule is required for autophosphorylation activity [46]. However, the kinase domain alone of numerous plant receptor-like kinases indeed show autophosphorylation activity *in vitro* [47].

One of the ways to decipher the function of a molecule is to generate a knockout cell line or to block expression of corresponding gene. Gene knockouts in *E. histolytica* have not been possible because of high ploidy of this organism [48]. However, it is possible to modulate the expression by antisense cloning. Since the gene may be essential for proliferation, a tetracycline inducible system was used. To establish the role of EhTMKB1-9, two approaches were used, one was to over express the RNA in antisense direction and the other was a dominant negative approach where a TMK molecule lacking the kinase domain was over-expressed so as to restrict signal transduction to the downstream molecules. Defects in cellular proliferation and target cell killing were observed for the transfected cell lines in both the approaches, emphasizing the fact that EhTMKB1-9 is an essential cell surface molecule. The dominant negative mutant highlights the importance of a functional kinase domain, as the over-expressed mutant could still bind ligand and compete with endogenous full length molecule or form a nonfunctional heterodimer with the wild-type molecule and thereby blocking the downstream signaling.

In conclusion, the results presented here show that EhTMKB1-9 is a transmembrane kinase that is likely to be involved in cellular proliferation and parasite virulence. It belongs to a family of TMKs whose members are differentially expressed in response to growth conditions. Currently efforts are being made to understand the signaling process initiated by serum and the mode of regulation of the TMKs.

## Materials and Methods

### Sequence analysis

The genes were identified in the 12.5× assembly available from The Institute for Genomic Research (TIGR) and Sanger sequencing centers (http://www.tigr.org/tdb/e2k1/eha1 and http://www.sanger.ac.uk/Projects/E_histolytica/) and NCBI by searching the database for homologs of XM_001913432. All of the hits that showed ≥95% sequence identity at the nucleotide level against XM_001913432 over a minimum stretch of 500 nucleotides were extracted. All the members were also searched in *Entamoeba* pathema database (http://pathema.jcvi.org/cgi-bin/Entamoeba/PathemaHomePage.cgi). Genescan was used to confirm the predicted coding regions of EhTMKB1 members. Conserved domains and motifs were identified by CD-search (http://www.ncbi.nlm.nih.gov/BLAST) and Pfam (http://pfam.sanger.ac.uk/search). The determination of amino acid composition and multiple alignments (CLUSTALW) were performed using the BioEdit sequence alignment editor (version 7.0; Tom Hall). Secondary structure, topology, and membrane organization were predicted by using the set of tools available at www.expasy.ch.

### Phylogenetic analysis of the TMKs

The *E. histolytica* TMK sequences were downloaded from the NCBI database using the locus tag defined by Beck *et al.* [12]. The TMKs were searched in NCBI and Pathema database of *E. dispar* and *E. invadens* for corresponding orthologs. In some cases, different EhTMKs mapped to the same orthologs of *E. dispar* or *E. invadens*. In these cases, the member which had maximum match length and score was considered as true ortholog. The accession numbers can be found in Table S3. The kinase domain sequence was extracted and used as input on phylogeny.fr platform for tree construction. MUSCLE was used for multiple alignment, Gblocks for automatic alignment curation and PhyML for tree building [49]. The tree drawing was done on dendroscope [50].

### Strains and cell culture

All experiments were carried out with *E. histolytica* strain HM-1:IMSS clone 6.The cells were maintained and grown in TYI-33 medium supplemented with 15% adult bovine serum, 1× diamond's vitamin mix and antibiotics (0.3 units/ml penicillin and 0.25 mg/ml streptomycin) at 35.5°C [51]. To achieve serum starvation condition, medium from mid log phase grown *Entamoeba* cells was replaced with TYI-33 medium containing 0.5% adult bovine serum for indicated time period. Serum replenishment was achieved by decanting the medium after 24 h of serum starvation and replacing with complete medium for indicated time period. G-418 or hygromycin B (Sigma) was added at 10 µg/ml for maintaining the transfected cell lines.

### Northern hybridization

Total RNA was purified using TRIzol reagent (Invitrogen) according to the manufacturer's instructions. RNA samples (30 µg) were resolved in formaldehyde agarose in gel running buffer [0.1 M MOPS (pH 7.0), 40 mM sodium acetate, 5 mM EDTA (pH 8.0)] and 37% formaldehyde at 4 V/cm. The RNA was transferred on to GeneScreen plus (NEN) nylon membranes. Hybridization and washing conditions for RNA blots were as per manufacturer's protocol.

### Reverse transcription (RT-PCR)

DNase I treated total RNA (5 µg) was used in the RT reaction using Superscript III (Invitrogen) with oligo dT primer. Annealing was carried out at 65°C for 5 min, followed by extension at 42°C for 1 h followed by inactivation at 70°C for 10 min. 1 µl of this RT mix was used for a regular PCR. PCR was performed with KDRTFP 5′-CCACTTGATGATAACATTGAAGTTAATTGTAAATTAAC-3′ and KDRTRP 5′-GCGCTCTAACATTCTTACAATTTCATCTATTGTTATT-3′ and amplification conditions were as follows: 94°C for 5 min; then 30 cycles at 94°C for 30 s, 48°C for 1 min and 72°C for 1 min; and a final extension at 72°C for 10 min. The PCR products were cloned in pGEMT-Easy vector (Promega). No amplicon was observed in the absence of RT enzyme in all RT-PCR experiments.

### Real Time PCR primer design

Real-time PCR primers (Table 1) were designed using Primer Express 3.0 (Applied Biosystems). Each primer was analyzed against the *E. histolytica* database and any primer that had significant sequence similarity to multiple genes was rejected. Thus, both the forward and reverse primers were specific for one gene, except FL-unique and kinase domain, which detected majority of family members in the genome. Optimal annealing conditions were used to ensure specificity and any PCR primer pair that produced more than one melt peak was discarded. PCR products were analyzed by gel electrophoresis in 1.5% agarose–Tris-borate-EDTA, and if multiple bands were observed, the primer pair was discarded.

### Quantitative Real Time (qRT-PCR)

Real time PCR efficiencies for each gene were calculated from the slope, according to the established equation E = 10^[−1/slope]^ using genomic DNA as template (serial 1∶10 fold dilutions) and were found around 1.96±0.06 [19]. Two µg total RNA (DNase I treated) was reverse transcribed using random hexamers into cDNA by Superscript III reverse transcriptase (Invitrogen). Real-time quantitative PCR was performed in 7500 Real Time PCR System (Applied Biosystems) using SYBR green PCR Master Mix, 2 pmol of forward and reverse primers and 2 µl of cDNA (serial 1∶10 fold dilution). EhTMKB1 members and the RNA Pol II (control gene) were amplified in parallel. The conditions were pre-denaturation at 95°C for 10 min, followed by 40 cycles at 95°C for 15 sec and 58°C for 1 min followed by a dissociation stage at 95°C for 15 sec and 58°C for 1 min. Cycle threshold values (*C*_t_) were analyzed by the SDS1.4 software (Applied Biosystems) and all samples were analyzed in triplicates in three independent experiments. Reactions without cDNA were used as no template control and no RT controls were also set up to rule out genomic DNA contamination. Relative quantification of EhTMKB1 expression was determined using the comparative *C*_t_ method (ABI Prism 7500, SDS User Bulletin; Applied Biosystems).

### Primer extension

DNase I treated total RNA (5 µg) was incubated with [γ-^32^P] labeled oligonucleotide 5′-CTGTCTGTACTACAACCAGAACTAC-3′ complementary to the nucleotides at positions 107 to 131 downstream to the start codon of EhTMKB1-9. Annealing was carried out at 65°C for 5 min, followed by extension at 42°C for 1 h with 200 U of Superscript III reverse transcriptase (Invitrogen). The products were separated on denaturing 6% urea-polyacrylamide gels, together with the sequencing reaction using the same oligonucleotide.

### RNase protection assay

278 bp fragment corresponding to −111 to +147 of EhTMKB1-9 gene was amplified using primers; TMK9F2 5′-CATGTGAATGAAAACAAAAACATAGTTG-3′ and T7T9RP 5′- TAATACGACTCACTATAGGGAGAGTCTGTACTACAACCAGAACTAC-3′. The purified fragment was transcribed in antisense orientation in the presence of 20 µCi of [α-^32^P] UTP using the T7 polymerase (MAXIscript *in vitro* transcription kit, Ambion). RNase protection assays were done with 30 µg of total RNA using the RPA III ribonuclease protection assay kit (Ambion). Briefly, RNA was hybridized with 80,000 counts of radiolabeled probe overnight at 42°C and digested with a mixture of RNase A and RNase T1 for 30 min at 37°C. The protected fragments were precipitated and analyzed on a denaturing 6% urea- polyacrylamide gel. 30 µg of yeast RNA was used as positive control for the function of RNase, and another sample containing the same amount of yeast RNA was incubated without RNase as a control for probe integrity. Primers used for RNase protection assay for EhActin were Actin RPAFP 5′-CAAGAAAATTAGCTAAGAAATTAAAATG-3′ and Actin RPAT7 RP 5′-TAATACGACTCACTATAGGGAGACGTGTCTTGGTCTACCAACAATGG-3′.

### 3′- RACE

DNase I treated total RNA (5 µg) was used in the RT reaction using Superscript III (Invitrogen) with oligo dT adapter 5′-GTGAGGGTACCTCTAGACTCGAGTTTTTTTTTTTTTTTTTT-3′. Annealing was carried out at 65°C for 5 min, followed by extension at 42°C for 1 h followed by inactivation at 70°C for 10 min. 2 µl of this RT mix was used for a regular PCR. PCR was performed with KDRTFP and adapter 5′-GTGAGGGTACCTCTAGACTCGAG-3′. Amplification conditions were as follows: 94°C for 5 min; then 30 cycles at 94°C for 30 s, 48°C for 1 min and 72°C for 1 min; and a final extension at 72°C for 10 min. The PCR products were cloned in pGEMT-Easy vector (Promega). No amplicon was observed in the absence of RT enzyme in all RT-PCR experiments.

### Luciferase reporter constructs

To facilitate cloning, restriction enzyme sites *Sac*I, *Sac*II and *Xho*I sites were introduced in the vector pEh-Neo-luc at the junction of 3′-actin and 5′-lectin with primers Site FP 5′-ATGGAGATGAAGACCGCGGAGCTCGAGTCATCCTGTTT-3′ and Site RP 5′-AAACAGGATGACTCGAGCTCCGCGGTCTTCATCTCCAT-3′ using site directed mutagenesis and the construct obtained was termed as pEh-Neo-luc-S (plectin). EhTMKB1-9 promoter fragment −939 to +15 was cloned in plectin by excising the 5′- lectin promoter region with *Xho*I and *Kpn*I and was replaced with PCR product obtained using the T9PrFP 5′-GCGCTCGAGAAGTTTTTAATACATAATTTATTC-3′ and T9PrRP 5′-GCGGGTACCTTCTAGTTCCAGTATTTAAGTAT-3′. Similarly, 920 nucleotides upstream to ORF of EhTMKB1-5 (a full length member) were cloned and construct termed as p5–920 using the primers T5PrFP 5′-CCGCTCGAGGAATAAACAATAAAAATAAATAG-3′ and T5PrRP 5′-GCGGGTACCAAATGAGTATGAAGAATAACAAT-3′. A promoter-less construct (pless) was also made by removing the 5′- lectin promoter region. All 5′- deletion constructs were PCR amplified with *Xho*I site in the forward primer at the desired position and using T9PrRP as reverse primer. All the fragments were PCR amplified from genomic DNA and inserted at *Xho*I and *Kpn*I sites upstream of luciferase gene. The orientation and sequence of each construct was confirmed by restriction digestion and DNA sequence analysis. The *Xho*I and *Kpn*I sites are underlined in the primers.

### Transfection and selection of *E. histolytica* trophozoites

Transfection was performed by electroporation as described previously [52]. Briefly, trophozoites in log phase were harvested and washed with PBS followed by incomplete cytomix buffer (10 mM K_2_HPO_4_/KH_2_PO_4_ (pH 7.6), 120 mM KCl, 0.15 mM CaCl_2_, 25 mM HEPES (pH 7.4), 2 mM EGTA, 5 mM MgCl_2_). The washed cells were then re-suspended in 0.8 ml of complete cytomix buffer (incomplete cytomix containing 4 mM adenosine triphosphate, 10 mM glutathione) containing 200 µg of plasmid DNA and subjected to two consecutive pulses of 3000 V/cm (1.2 kV) at 25 µF (Bio-Rad, electroporator). The transfectants were initially allowed to grow without any selection. Drug selection was initiated after 2 days of transfection in the presence of 10 µg/ml G-418 for constructs with luciferase reporter gene or 10 µg/ml of hygromycin B was used for tetracycline inducible constructs.

### Luciferase assay

The procedure was done as described previously [53]. Briefly, stably transfected trophozoites, maintained in TYI-S-33 medium supplemented with 10 µg/ml G-418, were chilled on ice, harvested and washed once in PBS (pH 7.4), and lysed in 200 µl of reporter lysis buffer (Promega) with the addition of protease inhibitors E64-C and leupeptin. Lysates were frozen overnight at −80°C. After thawing on ice for 10 min, cellular debris was pelleted, and the samples were allowed to warm to room temperature. Luciferase activity was measured according to the manufacturer's instructions (Promega) using a Turner Luminometer (model TD-20E). Luciferase activity per µg of protein was calculated as a measure of reporter gene expression.

### Expression and purification of recombinant EhTMKB1-9 kinase domain

EhTMKB1-9 kinase domain region (3123–3927 nucleotide position) was cloned in *BamH*I site of pET21a vector (Novagen). The primers, T9KdBamFP 5′-GCGGGATCCATGATAAAAGAAGAAAAGAAAATAGG-3′ and T9hisBam 5′-CTTGGATCCTTAATGATGATGATGATGATGTGATCCTCTATTATTACTGTAAACTGTTTCTAACAT-3′ were designed to amplify the kinase domain region of EhTMKB1-9 with a penta-his tag. A kinase dead site directed mutant was generated by mutating lysine at amino acid position 1069 to alanine with primers T9 K1069A FP 5′-GAAATAAAGTAGCAATTGCAAAAATGAAACAAATTG-3′ and T9 K1069A RP 5′-CAATTTGTTTCATTTTTGCAATTGCTACTTTATTTC-3′. Expression constructs were used to transform C43 *Escherichia coli* cells. The cells were grown overnight at 30°C in 10 ml of luria broth medium containing ampicillin. 2% inoculum of overnight culture was used as starter for a fresh 200 ml culture. This culture was incubated at 30°C with agitation for 2 h and then was transferred to 20°C for 1 h. The culture was induced by the addition of 0.3 mM isopropyl-β-D-thiogalactopyranoside (IPTG) followed by a further 10 h of incubation under the same conditions. The recombinant enzyme was extracted and purified on His-Select HF Nickel Affinity Gel (Sigma) as described by the manufacturer. The protein was dialysed against 20 mM HEPES pH 7.4 and 100 mM NaCl with two changes at interval of 2 h and was stored at 4°C.

### *In vitro* kinase assay

Purified recombinant T9 KD or T9 KD K1069A (0.5 µg) were used in a 30 µl reaction containing 20 mM HEPES pH7.4, 2.5 mM MgCl_2_, 2.5 mM MnCl_2_, 1× protease inhibitor cocktail, 1× phosphatase inhibitor cocktail I and II (Sigma) and 10 µg of histone Type III-S (Sigma) or 2 µg of myelin basic protein, MBP (Sigma). The protein kinase inhibitors genisten (10 µg/ml) and staurosporine (50 nm) were used as wherever indicated. The kinase reaction was initiated with addition of 0.5 µCi of [γ-^32^P] ATP per reaction and were incubated at 30°C for 45 min. The reaction was stopped by addition of 5× Laemmli sample buffer followed by boiling for 5 min. The products were resolved on SDS-PAGE (12% for histone and 14% for MBP). Phosphorylation was detected by Phosphor Imager (Fujifilm) analysis.

### Production of anti EhTMKB1-9 antiserum

The peptide LYDYSKYKSVIIRFS (amino acids 51–65 of EhTMKB1-9) was synthesized conjugated to KLH and used to immunize New Zealand white rabbits (NEP, USA). Resultant serum was affinity purified, dialyzed against PBS and stored at −20°C until use.

### Western analysis

For immunodetection, samples were separated on 8% SDS-PAGE. The gel was then transferred on to a PVDF membrane and processed using standard methods. The antigens were detected with affinity-purified anti-EhTMKB1-9 (1∶1000) and with anti-rabbit HRPO (1∶10000, Sigma). ECL reagents were used for visualization (Millipore). Actin was detected using anti-EhActin antibodies (raised in our laboratory) at 1∶1000 dilution.

### Immunofluorescence staining

Immunofluorescence staining was carried out as described before [54]. Briefly *E. histolytica* cells resuspended in TYI-33 medium were transferred onto acetone-cleaned coverslips placed in a petri dish and allowed to adhere for 10 min at 35.5°C. The culture medium was removed and cells were fixed with 3.7% pre-warmed paraformaldehyde (PFA) for 30 min. After fixation, the cells were permeabilized with 0.1% Triton X-100/PBS for 1 min. This step was omitted for non-permeabilized cells. The fixed cells were then washed with PBS and quenched for 30 min in PBS containing 50 mM NH_4_Cl. The coverslips were blocked with 1% BSA/PBS for 30 min, followed by incubation with primary antibody at 37°C for 1 h. The coverslips were washed with PBS followed by 1% BSA/PBS before incubation with secondary antibody of 30 min at 37°C. Antibody dilutions used were: anti-EhTMKB1-9 at 1∶20, anti-rabbit Alexa 488 (Molecular Probes) at 1∶300. The preparations were further washed with PBS and mounted on a glass slide using DABCO [1,4-diazbicyclo (2,2,2) octane (Sigma) 10 mg/ml in 80% glycerol]. The edges of the coverslip were sealed with nail-paint to avoid drying. Confocal images were visualized using an Olympus Fluoview FV1000 laser scanning microscope.

### EhTMKB1-9 gene construct in sense and antisense orientation

The CAT gene was excised from pEhHYG-tetR-O-CAT (TOC) vector with *Kpn*I and *BamH*I restriction endonucleases and EhTMKB1-9 sense, dominant negative mutant and antisense PCR products were inserted in desired orientation. The primers used were TMK9FP 5′- TATGGTACCATGTCATTTATGACATATTATTTTTTATTGCTG-3′ and TMK9RP 5′-CTTGGATCCTTAATGATGATGATGATGATGTGATCCTCTATTATTACTGTAAACTGTTTCTAACAT-3′ for cloning EhTMKB1-9 in sense orientation while T9BamFP 5′-GCGGATCCATGTCATTTATGACATATTATTTTTTA-3′ and T9Kpn1000RP 5′-CTTGGTACCTGTTGAAATTGACTTGTCACTATC-3′ were used for cloning 1.0 kb ORF region from N-terminal of EhTMKB1-9 in anti-sense orientation. TMK9FP and TMK9DnRP 5′- CTTGGATCCTTAATGATGATGATGATGATGTGATCCTCTAAGTCGTGTTGATATTTC-3′ primers were used to amplify and clone dominant negative mutant (TMK9-Dn). The restriction enzyme sites are underlined.

### Cellular proliferation

*E. histolytica* cell transfectants TOC, TMK9-S, TMK9-Dn, TMK9-AS were grown in the presence of 20 µg/ml hygromycin B, and growth was measured in the absence and presence of 10 µg/ml tetracycline after the indicated time periods. The cells were counted by haemocytometer. Cell viability was determined by microscopy using trypan blue dye exclusion test.

### Adhesion and cytopathic activity on cell monolayers

Adhesion assay was performed as described by Padilla-Vacca *et al* [55]. Monolayers of cultured CHO cells were grown in Dulbecco's modified Eagle's medium (DMEM) with 10% fetal calf serum (FCS) to confluence in 24-well plates. For adhesion assays, the monolayer was washed once with DMEM followed by PBS and was fixed with 4% formaldehyde for 30 min. The fixed cells were washed twice with PBS, incubated with a solution of glycine (250 mM) for 30 min, and washed twice with PBS. Transfectant trophozoites (2×10^5^) were added to wells containing fixed monolayers in 1 ml of DMEM without serum and incubated at 37°C for 30 min. The number of parasites adherent to CHO cells was determined by counting the trophozoites that remained adhered to the cell monolayer after gentle decantation (two times) of the nonadhered trophozoites with warm DMEM. The number of trophozoites that remained adhered to CHO monolayer for HM1 was taken as 100%. For cytopathic-activity assays, the rate of destruction of the CHO cell monolayer by transfected trophozoites was evaluated as previously described by Bracha and Mirelman [56]. Briefly, trophozoites (2×10^5^ ml^−1^ suspended in DMEM without FCS) were added in triplicate to wells containing a confluent monolayer of CHO cells (2×10^5^ ml^−1^) pre-washed with DMEM to remove traces of fetal calf serum and incubated for 60 min at 37°C in an atmosphere of 95% air and 5% CO_2_. The reaction was stopped by chilling for 10 min and the wells were then washed thrice with cold PBS. The monolayer was fixed with 4% paraformaldehyde for 10 min and stained with methylene blue (0.1% in borate buffer, 0.1 M, pH 8.7). The excess stain was washed with 0.01 M borate buffer and the incorporated dye was extracted by adding 1.0 ml of 0.1 M HCl at 37°C for 30 min. The colour was read in a spectrophotometer at 660 nm after appropriate dilutions with 0.1 M HCl. Destruction of cells was expressed in relation to the amount of dye extracted from the control monolayer of CHO cells. The percentage of monolayer destruction by HM1 was taken as 100%.

### Miscellaneous methods

The concentration of protein in a sample was estimated by bicinchoninic acid assay using BSA as a standard. SDS-PAGE analysis was carried out in 12%–14% acrylamide gels under reducing conditions according to the method of Laemmli [57].

### Accession numbers of EhTMKB1s

EhTMKB1-1, XM_001913432; EhTMKB1-2, XM_001914145; EhTMKB1-3, XM_646349; EhTMKB1-4, XM_001914305; EhTMKB1-5, XM_645102; EhTMKB1-6, XM_001914353; EhTMKB1-7, XM_649257; EhTMKB1-8A, XM_001914177; EhTMKB1-8B, XM_001914176; EhTMKB1-9, XM_648866; EhTMKB1-10, XM_649448; EhTMKB1-11, XM_643752; EhTMKB1-12, XM_001914281; EhTMKB1-13, XM_001914190; EhTMKB1-14, XM_645177; EhTMKB1-15, XM_646773; EhTMKB1-16, XM_650136; EhTMKB1-17A, XM_001914383; EhTMKB1-17B, XM_647245; EhTMKB1-18A, EHI_123290; EhTMKB1-18B, XM_001914168; EhTMKB1-19, XM_001913662; EhTMKB1-20, XM_001914098; EhTMKB1-21, XM_001914214; EhTMKB1-22, XM_00191404**7**; EhTMKB1-23, XM_001914010; EhTMKB1-24, XM_001914144; EhTMKB1-25, XM_001914041; EhTMKB1-26, XM_646820; EhTMKB1-27, XM_001914011; EhTMKB1-29, XM_645087; EhTMKB1-30, XM_001914494; EhTMKB1-31, XM_001914477; EhTMKB1-32, XM_643685; EhTMKB1-33,XM_001914166; EhTMKB1-34, XM_001913644; EhTMKB1-35, XM_001914563.

## Supporting Information

Figure S1 **EhTMKB1 family of transmembrane kinases of** ***E. histolytica*** **.** (A) The functional domain organization of a typical full length EhTMKB1 member based on amino acid composition is indicated schematically. Signal peptide sequence (SP), asparagine-rich region (Asn rich), cysteine-rich motifs (Cys rich), trans-membrane region (TM), linker and kinase domain. (B) The members were identified on the basis of ≥95% sequence identity at nucleotide level with a full length member EhTMKB1-1 (XM_001913432). The putative ORFs are shown in colored boxes while the non coding matched regions are shown in black lines. Some of the members have ORF regions that do not have similarity with EhTMKB1 full length member and are boxed and shown in different colours. Putative introns are indicated by yellow lines and * denotes the contig end. EhTMKB1-9 and 18 are marked with green and pink arrows. Detailed information of EhTMKB1 members (accession number and other features) is in Table S1.
**Explanation:**
Out of 28 EhTMKB1 members defined by Mehra *et al* 2006, 14 locus tags have been discontinued as the *Entamoeba histolytica* genome has been re-annotated. Since the *E. histolytica* genome database has been updated, with improved assembly, we repeated the database search to update the list of members belonging to EhTMKB1 family and information is schematically presented. The analysis identified 35 members with only six full length members. New introns have been detected and stop codons have been removed for some EhTMKB1 members. The sequence of each member has been manually checked and all the major differences, accession numbers and earlier nomenclature used are listed in Table S1.
The following information is not present in the Figure 1 of Mehra *et al* 2006, and is new information obtained in the present analysis.The N-terminal part of the ORF of a few EhTMKB1 members (EhTMKB1-7, 9, 10 and 11 - boxed region and shown in different color with respect to full length member) is unique to that particular member. This unique sequence is expressed in each case and was confirmed by RT-PCR. Thus, the N-terminal region of these copies may have been acquired after the duplication or the expansion event took place in the gene-family.The Figure S1 shows the coding (colored boxes) and non coding (black lines) regions of EhTMKB1 members. The ORFs of some members, such as EhTMKB1-13, 16, 19-28 and others were found to be much shorter than the entire matched stretch of the sequence.Putative introns (yellow lines) and contig ends (*) have been marked.The C- terminal ends of eight EhTMKB1 members are different with respect to the full length members (grey colored boxes).Three members were predicted to code for two annotated genes within the matched conserved region (EhTMKB1-8A/8B, EhTMKB1-17A/17B and EhTMKB1-18A/18B).
(0.17 MB TIF)

Figure S2 **Southern hybridization to confirm the predicted organization of EhTMKB1 members.** (A) Schematic representation of EhTMKB1 full length member (refer to Figure S1), indicated contigs and the position of restriction enzymes used in study. Open reading frames (ORFs) are shown in colour. $ - The contig sequence is reverse complemented as ORF is in opposite direction and the positions are shown accordingly. (B) Southern hybridization of digested genomic DNA using probes which is specific for EhTMKB1 members (probe region used are highlighted in color - dark blue for lanes 1–4, dark green for lane 5, light pink for lane 6). Lanes 1–6 represent contigs 1–6 of panel A. (C) Hybridization was carried out with a probe derived from conserved kinase domain. Restriction enzymes sites used are; lane 1, *Sca*I + *Nco*I; lane 2, *Hind*III + *Hinc*II; lane 3, *Pac*I; lane4, *Xmn*I; lane 5, *Sca*I + *Nco*I; lane 6, *EcoR*I + *Nco*I and the expected sizes of bands are 4765, 7487, 4839 and 5553, 5630, 4394, 3183 bp for lane 1–6 respectively. The probe used for lane 5 is specific for EhTMKB1-9 and the same as described in Figure 6. The probe used for lane 6 is specific for EhTMKB1-18 and the same as described in Figure 5 (probe II).The restriction enzyme positions for selected EhTMKB1 members are given in Table S2.
**Explanation:**
The results obtained for southern hybridization of EhTMKB1 members were in agreement with predicted genome assembly. For example, the probe derived from EhTMKB1-9 (Figure S2B, lane 5) and EhTMKB1-18 (Figure S2B, lane 6) gave bands of 4.3 and 3.2 kb similar to the expected size. The probe derived from full length members, showed expected sizes along with other sizes in lane 1–4. This was expected as the probe (unique - blue color region; see Figure S1) was derived from region shared by sequences of six full length members and four 3′- truncated members which lacks the kinase domain. Since kinase domain was part of most of the members hybridization with a probe derived from kinase domain generated multiple bands (Figure S2C).
(0.40 MB TIF)

Figure S3 **Multiple alignment of representative sequences obtained from different libraries.** Minor differences in sequences are highlighted that help to identify the origin of these sequences.
(0.16 MB TIF)

Table S1 **Detailed analysis of EhTMKB1 members.**
(0.09 MB DOC)

Table S2 **The position of restriction enzymes used in southern hybridization of selected EhTMKB1 members.**
(0.03 MB DOC)

Table S3 **Accession number of** ***Entamoeba*** **transmembrane kinases used for phylogenetic analysis.**
(0.19 MB DOC)

Table S4 **Comparison of kinase subdomains of EhTMKB1 members as per Hank's classification.**
(0.07 MB DOC)
