# Supplementary figures and images for: Serum-Dependent Selective Expression of EhTMKB1-9, a Member of Entamoeba histolytica B1 Family of Transmembrane Kinases
Source: PLoS Pathog. 2010 Jun 3;6(6):e1000929. doi: 10.1371/journal.ppat.1000929 (PMC2880585; doi:10.1371/journal.ppat.1000929)

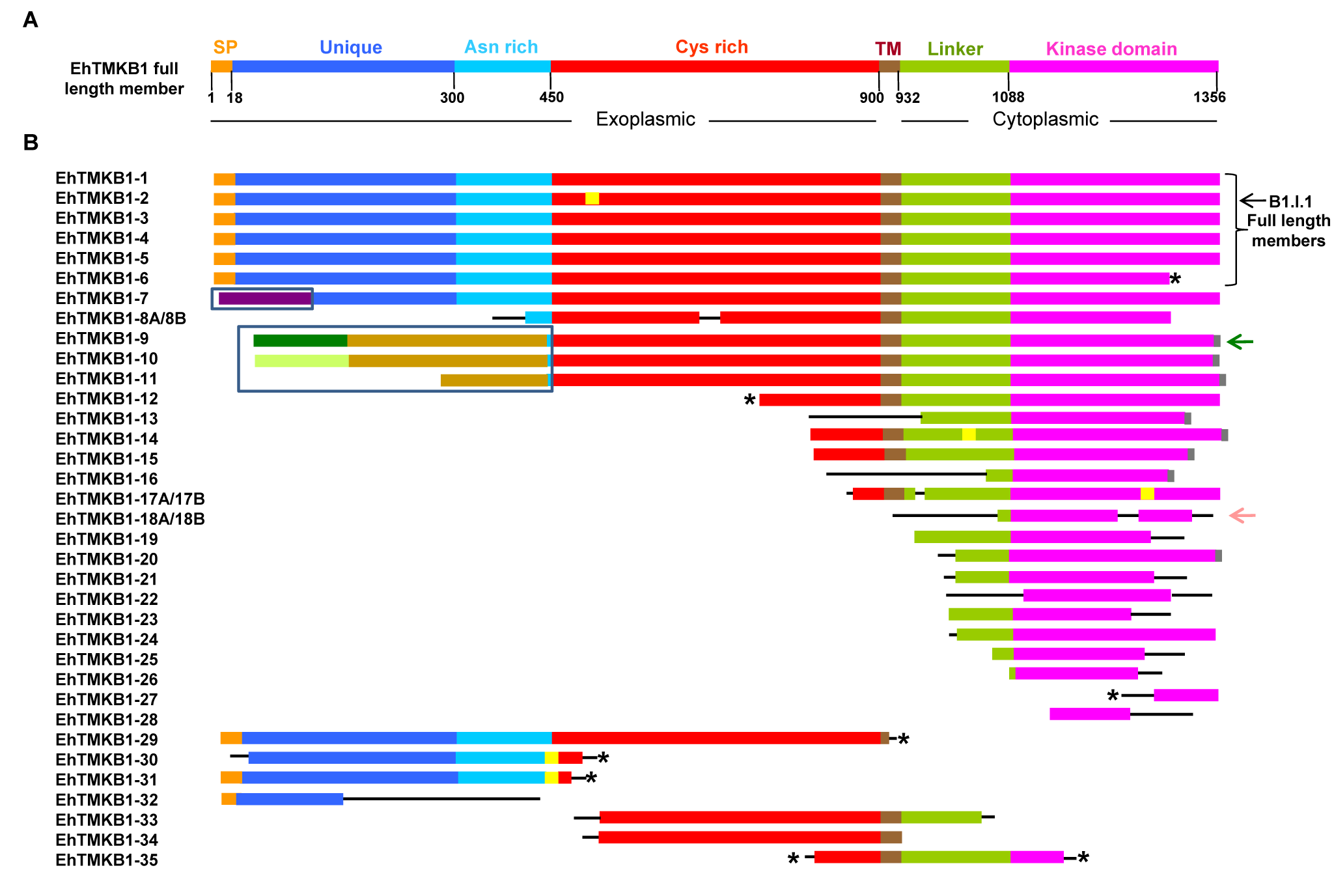

Supplement: Figure S1 — EhTMKB1 family of transmembrane kinases of E. histolytica . (A) The functional domain organization of a typical full length EhTMKB1 member based on amino acid composition is indicated schematically. Signal peptide sequence (SP), asparagine-rich region (Asn rich), cysteine-rich motifs (Cys rich), trans-membrane region (TM), linker and kinase domain. (B) The members were identified on the basis of ≥95% sequence identity at nucleotide level with a full length member EhTMKB1-1 (XM_001913432). The putative ORFs are shown in colored boxes while the non coding matched regions are shown in black lines. Some of the members have ORF regions that do not have similarity with EhTMKB1 full length member and are boxed and shown in different colours. Putative introns are indicated by yellow lines and * denotes the contig end. EhTMKB1-9 and 18 are marked with green and pink arrows. Detailed information of EhTMKB1 members (accession number and other features) is in Table S1. Explanation: Out of 28 EhTMKB1 members defined by Mehra et al 2006, 14 locus tags have been discontinued as the Entamoeba histolytica genome has been re-annotated. Since the E. histolytica genome database has been updated, with improved assembly, we repeated the database search to update the list of members belonging to EhTMKB1 family and information is schematically presented. The analysis identified 35 members with only six full length members. New introns have been detected and stop codons have been removed for some EhTMKB1 members. The sequence of each member has been manually checked and all the major differences, accession numbers and earlier nomenclature used are listed in Table S1. The following information is not present in the Figure 1 of Mehra et al 2006, and is new information obtained in the present analysis.The N-terminal part of the ORF of a few EhTMKB1 members (EhTMKB1-7, 9, 10 and 11 - boxed region and shown in different color with respect to full length member) is unique to that particular membe [file ppat.1000929.s001.tif]

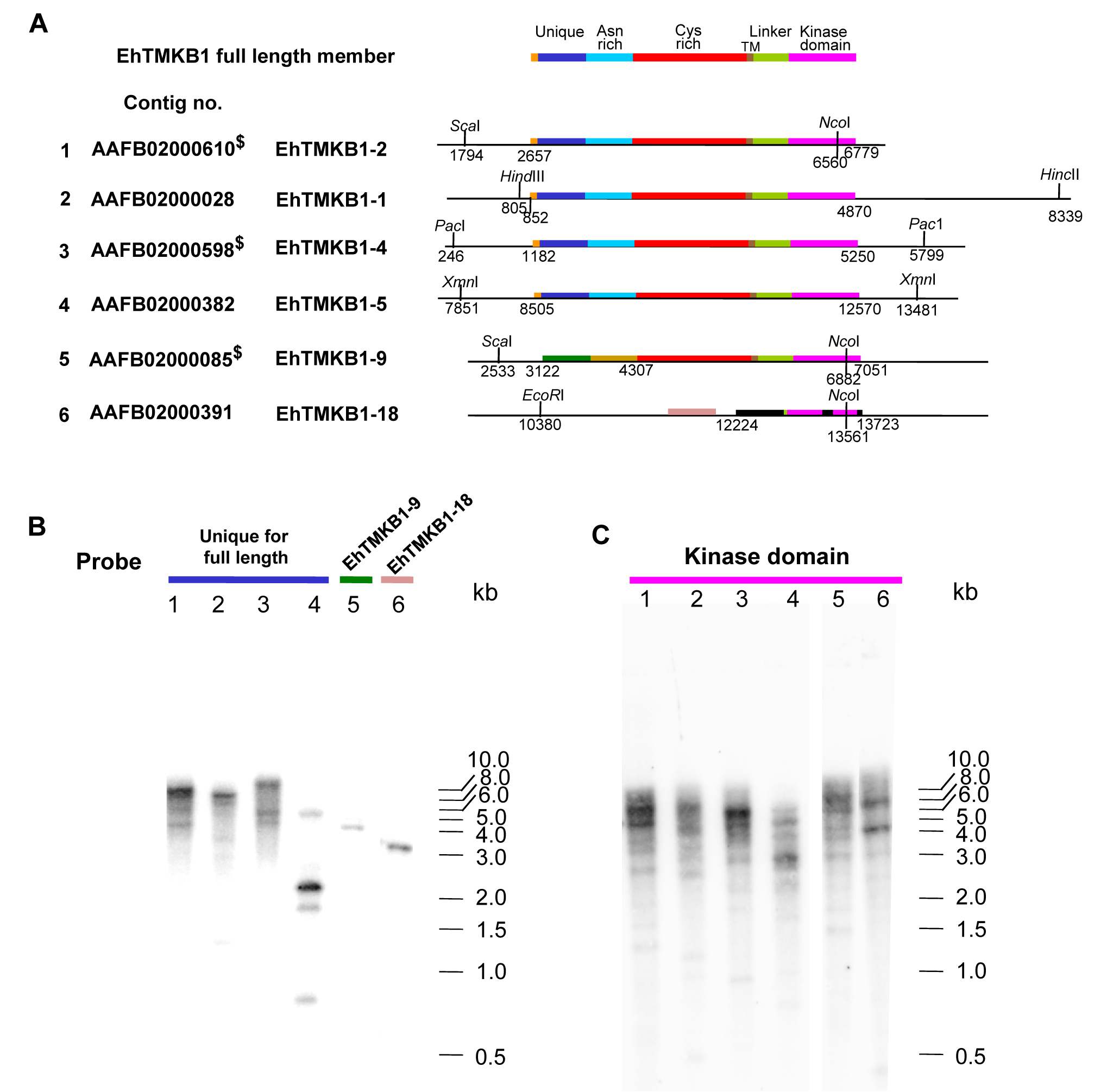

Supplement: Figure S2 — Southern hybridization to confirm the predicted organization of EhTMKB1 members. (A) Schematic representation of EhTMKB1 full length member (refer to Figure S1), indicated contigs and the position of restriction enzymes used in study. Open reading frames (ORFs) are shown in colour. $ - The contig sequence is reverse complemented as ORF is in opposite direction and the positions are shown accordingly. (B) Southern hybridization of digested genomic DNA using probes which is specific for EhTMKB1 members (probe region used are highlighted in color - dark blue for lanes 1–4, dark green for lane 5, light pink for lane 6). Lanes 1–6 represent contigs 1–6 of panel A. (C) Hybridization was carried out with a probe derived from conserved kinase domain. Restriction enzymes sites used are; lane 1, ScaI + NcoI; lane 2, HindIII + HincII; lane 3, PacI; lane4, XmnI; lane 5, ScaI + NcoI; lane 6, EcoRI + NcoI and the expected sizes of bands are 4765, 7487, 4839 and 5553, 5630, 4394, 3183 bp for lane 1–6 respectively. The probe used for lane 5 is specific for EhTMKB1-9 and the same as described in Figure 6. The probe used for lane 6 is specific for EhTMKB1-18 and the same as described in Figure 5 (probe II).The restriction enzyme positions for selected EhTMKB1 members are given in Table S2. Explanation: The results obtained for southern hybridization of EhTMKB1 members were in agreement with predicted genome assembly. For example, the probe derived from EhTMKB1-9 (Figure S2B, lane 5) and EhTMKB1-18 (Figure S2B, lane 6) gave bands of 4.3 and 3.2 kb similar to the expected size. The probe derived from full length members, showed expected sizes along with other sizes in lane 1–4. This was expected as the probe (unique - blue color region; see Figure S1) was derived from region shared by sequences of six full length members and four 3′- truncated members which lacks the kinase domain. Since kinase domain was part of most of the members hybridization with a probe derived from kinase domain [file ppat.1000929.s002.tif]

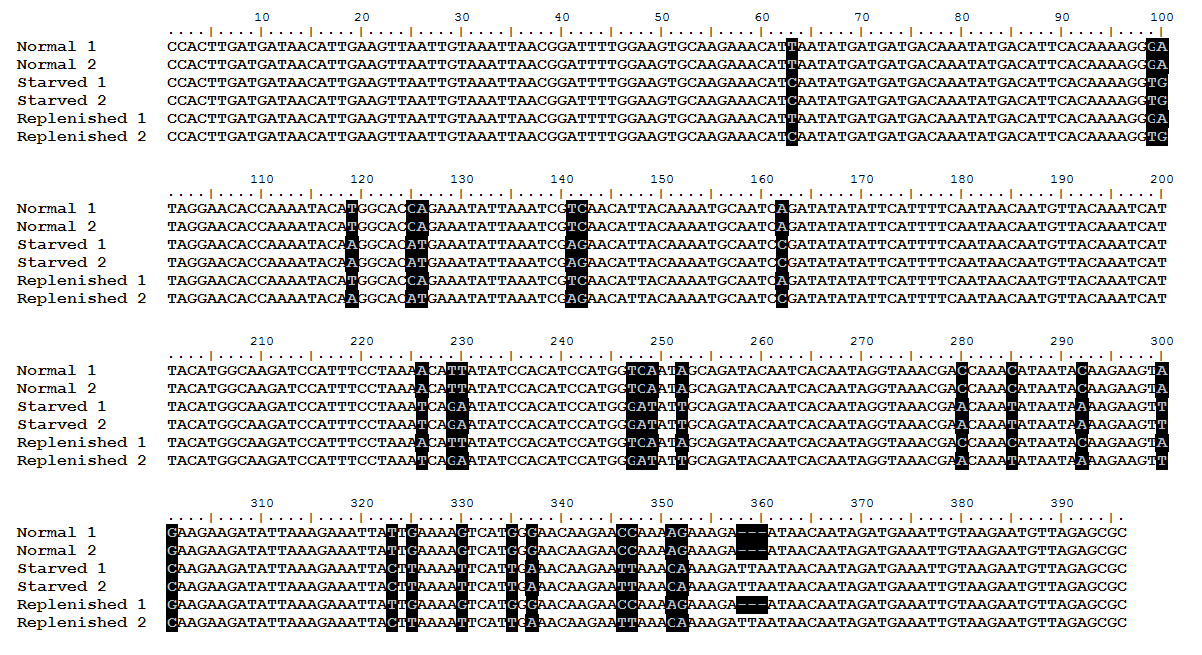

Supplement: Figure S3 — Multiple alignment of representative sequences obtained from different libraries. Minor differences in sequences are highlighted that help to identify the origin of these sequences. (0.16 MB TIF) [file ppat.1000929.s003.tif]
