# Supplementary material for: Serum-Dependent Selective Expression of EhTMKB1-9, a Member of Entamoeba histolytica B1 Family of Transmembrane Kinases
Source: PLoS Pathog. 2010 Jun 3;6(6):e1000929. doi: 10.1371/journal.ppat.1000929 (PMC2880585; doi:10.1371/journal.ppat.1000929)
Supplement: Table S1 — Detailed analysis of EhTMKB1 members. (0.09 MB DOC) [file ppat.1000929.s004.doc]

| **Table S1. Detailed analysis of EhTMKB1 members ^$^**. | | | | | | | | | | |
| --- | --- | --- | --- | --- | --- | --- | --- | --- | --- | --- |
| **EhTMKB1**  **member** | **Contig No.** | **Match region position** | **Match length** | **Pathema_ID** | **mRNA** | **ORF length** | **Protein** | **Class**  **member ^¥^** | **TMK No.*** | **Remarks** |
| EhTMKB1-1 | AAFB02000028 | 805-4872 | 4068 | EHI_103240 | XM_001913432 | 4068 | XP_001913467 | - | - | Full length member. |
| EhTMKB1-2 | AAFB02000610 | 4890-818 | 4073 | EHI_059040 | XM_001914145 | 4029 | XP_001914180 | B1.I.1 |  | Full length member and intron predicted at position 3461-3418. |
| EhTMKB1-3 | AAFB02000601 | 2670-6737 | 4068 | EHI_034210 | XM_646349 | 4068 | XP_651441 | B1.I.3 | TMK103 | Full length member. |
| EhTMKB1-4 | AAFB02000598 | 6608-2540 | 4071 | EHI_195370 | XM_001914305 | 4071 | XP_001914340 | - | - | Full length member. |
| EhTMKB1-5 | AAFB02000382 | 8505-12570 | 4068 | EHI_062090 | XM_645102 | 4068 | XP_650194 | B1.I.4 | TMK86 | Full length member. |
| EhTMKB1-6 | AAFB02000806 | 3834-1 | 3834 | EHI_128800 | XM_001914353 | 3786 | XP_001914388 | - | - | Full length member but contig ends at the 3´end, ORF is of 3834 nucleotides. |
| EhTMKB1-7 | AAFB02000251 | 20690-16999 | 3717 | EHI_120930 | XM_649257 | 4095 | XP_654349 | B1.I.6 | - | Initial 378 nucleotides of ORF do not match with EhTMKB1 conserved region. |
| EhTMKB1-8A  EhTMKB1-8B | AAFB02000409 | 13308-10589 | 2720 | EHI_157160/  EHI_157150 | XM_001914177/  XM_001914176 | 549/  1779 | XP_001914212/  XP_001914211 | - | - | 2 ORFs predicted in match region; one 13117 to 12569 and other is 12367-10589. |
| EhTMKB1-9 | AAFB02000085 | 43083-40362 | 2722 | EHI_073660 | XM_648866 | 3930 | XP_653958 | B1.II.4 | TMK95 | Initial 1186 nucleotides of ORF do not match with EhTMKB1 conserved region. |
| EhTMKB1-10 | AAFB02000195 | 15660-12948 | 2713 | EHI_092300 | XM_649448 | 3906 | XP_654540 | B1.II.2 | - | Initial 1171 nucleotides of ORF do not match with EhTMKB1 conserved region. |
| EhTMKB1-11 | AAFB02000326 | 3549-789 | 2713 | EHI_175920 | XM_643752 | 3255 | XP_648844 | B1.II.3 | TMK05 | Initial 520 nucleotides of ORF do not match with EhTMKB1 conserved region. |
| EhTMKB1-12 | AAFB02000597 | 7804-5933 | 1872 | EHI_001630 | XM_001914281 | 1872 | XP_001914316 | - | - | ORF starts with AGT as first codon, contig ends at the start of match region. |
| EhTMKB1-13 | AAFB02000427 | 11092-12597 | 1506 | EHI_185130 | XM_001914190 | 1080 | XP_001914225 | - | - | Initial 450 nucleotides of matched region are not the part of ORF and have stop codons in between. Predicted ORF position 12360 to 13407. |
| EhTMKB1-14 | AAFB02000184 | 932-2603 | 1672 | EHI_119250 | XM_645177 | 1635 | XP_650269 | B1.III.2 | - | Predicted intron position 1588 to 1649. |
| EhTMKB1-15 | AAFB02000159 | 4580-3092 | 1489 | EHI_011170 | XM_646773 | 1524 | XP_651865 | - | TMK12 | - |
| EhTMKB1-16 | AAFB02000120 | 36970-38439 | 1470 | EHI_136500 | XM_650136 | 726 | XP_655228 | - | - | Initial 765 nucleotides of matched region are not the part of ORF and have stop codons in between. Predicted ORF from 37735 to 38439. |
| EhTMKB1-17A  EhTMKB1-17B | AAFB02000894 | 1667-3302 | 1636 | EHI_066500/  EHI_066510 | XM_001914383/  XM_647245 | 327/  1167 | XP_001914418/  XP_652337 | - | - | Two ORFs predicted- one from 1678-2007 and another from 2089-3302 and an intron from 2988 to 3039. |
| EhTMKB1-18A  EhTMKB1-18B | AAFB02000391 | 12224-13723 | 1500 | EHI_123290/  EHI_123291 | na/  XM_001914168 | 579/  261 | na/  XP_001914203 | - | - | Initial 452 nucleotides of matched region are not the part of ORF. Two ORFs predicted, one is from 12676-13257 and other is from 13387 -13647 while match goes till 13723. (Prediction is wrong see Figure 5) |
| EhTMKB1-19 | AAFB02000162 | 31469-30375 | 1095 | EHI_062810 | XM_001913662 | 822 | XP_00191369 | - | - | 31397 -30608 of match region codes for ORF. |
| EhTMKB1-20 | AAFB02000310 | 13466-12274 | 1193 | EHI_123840 | XM_001914098 | 1074 | XP_001914133 | - | - | Initial 165 nucleotides of matched region are not the part of ORF but on translation shows exact similarity with EhTMKB1 full length member, only 13301 to 12228 codes for an ORF. |
| EhTMKB1-21 | AAFB02000460 | 10170-11170 | 1001 | EHI_057440 | XM_001914214 | 603 | XP_00191424 | - | - | Initial 165 nucleotides of matched region are not the part of ORF and 10336 to 10938 codes for an ORF. |
| EhTMKB1-22 | AAFB02000281 | 10550-11539 | 990 | EHI_172740 | XM_001914047 | 555 | XP_001914082 | - | - | Initial 295 nucleotides of matched region are not the part of ORF and have stop codon in between. 10846 to 11400 codes for an ORF. |
| EhTMKB1-23 | AAFB02000466 | 10792-11867 | 1076 | EHI_147730 | XM_001914010 | 612 | XP_001914045 | - | - | Initial 4 nucleotides (ATGA) of ORF do not match with conserved region of EhTMKB1; stop codon at 11400 position truncates the ORF while match goes till 11867. |
| EhTMKB1-24 | AAFB02000573 | 7973-6889 | 1085 | EHI_058930 | XM_001914144 | 1056 | XP_001914179 | - | - | Initial 29 nucleotides of match region are not the part of ORF, rest from 7943 to 6888 codes for ORF. |
| EhTMKB1-25 | AAFB02000274 | 8722-7839 | 884 | EHI_105820 | XM_001914041 | 426 | XP_001914076 | - | - | Initial 21 nucleotides of ORF do not match with conserved region of EhTMKB1, stop codon at 8295 position results into a truncated ORF while match goes till 8722. |
| EhTMKB1-26 | AAFB02000419 | 1092-1702 | 611 | EHI_082040 | XM_646820 | 492 | XP_651912 | - | - | Initial 3 nucleotides (ATG) of ORF do not match with conserved region of EhTMKB1, stop codon at 1581 position results into a truncated ORF while match goes till 1702. |
| EhTMKB1-27 | AAFB02000443 | 1-525 | 525 | EHI_147740 | XM_001914011 | 345 | XP_001914046 | - | - | Contig ends and initial 180 nucleotides of matched region are not the part of ORF. 181 to 525 codes for ORF. |
| EhTMKB1-28 | AAFB02000137 | 2285-1577 | 708 | - | na | - | - | - |  | Multiple stop codons. |
| EhTMKB1-29 | AAFB02000946 | 962-3730 | 2749 | EHI_116260 | XM_645087 | 2721 | XP_650179 | B.1.I.5 | TMK91 | Contig ends and stop codon at 3683 position results in truncated member while match goes till 3703. |
| EhTMKB1-30 | AAFB02001349 | 1498-20 | 1479 | EHI_109470 | XM_001914494 | 1176 | XP_001914529 | - | - | Contig ends and has intron from 199-145, ORF from 1316 to 86 while match further goes on till 20. |
| EhTMKB1-31 | AAFB02001264 | 1442-1 | 1442 | EHI_165920 | XM_001914477 | 1356 | XP_001914512 | - | - | Contig ends and has intron from 145-91, ORF from1442 to 32, while match goes on till 1. |
| EhTMKB1-32 | AAFB02000261 | 20189-21433 | 1209 | EHI_075730 | XM_643685 | 411 | XP_648777 | B.1.V.4 | - | Contig ends and stop codon at 20334 while match goes till 21433. |
| EhTMKB1-33 | AAFB02000385 | 14679-12951 | 1729 | EHI_160750 | XM_001914166 | 1683 | XP_001914201 | - | - | Initial 137 nucleotides of matched region are not the part of ORF, 14680-12951 codes for ORF and have 89 nucleotides extra at C-terminal. |
| EhTMKB1-34 | AAFB02000038 | 46901-48218 | 1318 | EHI_030860 | XM_001913644 | 1287 | XP_001913679 | - | - | Initial 32 nucleotides of matched region are not the part of ORF, 46933-48187 codes for ORF. |
| EhTMKB1-35 | AAFB02001768 | 1-1231 | 1231 | EHI_091840 | XM_001914563 | 1044 | XP_001914598 | - | - | Contig ends on both sides, Initial 64 nucleotides of matched region are not the part of ORF, 65 -1068 codes for ORF while match goes on till 1231. |

$- Refer to Figure S1 for alignment.

¥ - Classification nomenclature as used by Mehra *et al* 2006. Rest all members have been discontinued due to differences between and old and new annotated database (14 locus tags have been discontinued, new introns have been detected and stop codons have been removed)

*- Six EhTMKB1 members have been assigned TMK number by Beck *et al* 2005.

na- not available.
