# Supplementary material for: Serum-Dependent Selective Expression of EhTMKB1-9, a Member of Entamoeba histolytica B1 Family of Transmembrane Kinases
Source: PLoS Pathog. 2010 Jun 3;6(6):e1000929. doi: 10.1371/journal.ppat.1000929 (PMC2880585; doi:10.1371/journal.ppat.1000929)
Supplement: Table S2 — The position of restriction enzymes used in southern hybridization of selected EhTMKB1 members. (0.03 MB DOC) [file ppat.1000929.s005.doc]

**Table S2. The position of restriction enzymes used in southern hybridization of selected EhTMKB1 members**^*^**.**

| **S. No.** | **Contig No.** | **EhTMKB1 member** | **Match region**  **position** | **Match length** | ***EcoR*I** | ***Hind*III** | ***Hinc*II** | ***Nco*I** | ***Pac*I** | ***Sca*I** | ***Xmn*I** |
| --- | --- | --- | --- | --- | --- | --- | --- | --- | --- | --- | --- |
| **1** | **AAFB02000610^$^** | EhTMKB1-2 | 2657-6559 | 4073 | 2279 | 2704 | 4204 | 6560 | 6769 | 146, 1797 | 146, 369, 449, 673, 1528, 2010 |
| **2** | **AAFB02000028** | EhTMKB1-1 | 805-4872 | 4068 | - | 852 | 8339 | 4703 | - | - | 1138, 8398, 9693 |
| **3** | **AAFB02000598^$^** | EhTMKB1-4 | 1182-5250 | 4071 | - | 1229 | - | 5083, 6544 | 242, 246, 5799, 6318, 6344 | - | 1515, 4944, 6160 |
| **4** | **AAFB02000382** | EhTMKB1-5 | 8505-12570 | 4068 | 8121 | - | 2634, 5120, 12304 | 12403 | 7768,8049,  13664,14244 | 3423 | 1584, 2375, 4205, 7851, 13480 |
| **5** | **AAFB02000085^$^** | EhTMKB1-9 | 4307-6882 | 2722 | 13 | - | 6783, 10052 | 6882 | 4099 | 1899, 2531 | 4312 |
| **6** | **AAFB02000391** | EhTMKB1-18 | 12224-13723 | 1500 | 8553,  8766, 10379 | 6529 | 3040, 3797,  11488 | 13562 | 6654, 11252, 14038 | 5457, 11692 | 8650,8770, 9705, 10953, 11087 |

$ -full contig has been reverse complemented, *- restriction endonucleases positions are with respect to contig first nucleotide.
