# Supplementary material for: Serum-Dependent Selective Expression of EhTMKB1-9, a Member of Entamoeba histolytica B1 Family of Transmembrane Kinases
Source: PLoS Pathog. 2010 Jun 3;6(6):e1000929. doi: 10.1371/journal.ppat.1000929 (PMC2880585; doi:10.1371/journal.ppat.1000929)
Supplement: Table S3 — Accession number of Entamoeba transmembrane kinases used for phylogenetic analysis. (0.19 MB DOC) [file ppat.1000929.s006.doc]

**Table S3. Accession number of *Entamoeba* transmembrane kinases used for phylogenetic analysis.**

***E. histolytica*** TMKs

| **EhTMK** | **Pathema_ID** | **mRNA** | **Protein** |
| --- | --- | --- | --- |
| EhTMKA-4 | EHI_068720 | XM_645410 | XP_650502 |
| EhTMKA-17 | EHI_197350 | XM_646584 | XP_651676 |
| EhTMKA-23 | EHI_057680 | XM_647001 | XP_652093 |
| EhTMKA-52 | EHI_148550 | XM_652392 | XP_657484 |
| EhTMKA-53 | EHI_012220 | XM_651094 | XP_656186 |
| EhTMKA-61 | EHI_184540 | XM_644589 | XP_649681 |
| EhTMKA-65 | EHI_118810 | XM_649022 | XP_654114 |
| EhTMKA-69 | EHI_114160 | XM_646673 | XP_651765 |
| EhTMKA-85 | EHI_128430 | XM_646419 | XP_651511 |
| EhTMKB1-1 | EHI_103240 | XM_001913432 | XP_001913467 |
| EhTMKB1-2 | EHI_059040 | XM_001914145 | XP_001914180 |
| EhTMKB1-3 | EHI_034210 | XM_646349 | XP_651441 |
| EhTMKB1-4 | EHI_195370 | XM_001914305 | XP_001914340 |
| EhTMKB1-5 | EHI_062090 | XM_645102 | XP_650194 |
| EhTMKB1-7 | EHI_120930 | XM_649257 | XP_654349 |
| EhTMKB1-9 | EHI_073660 | XM_648866 | XP_653958 |
| EhTMKB1-10 | EHI_092300 | XM_649448 | XP_654540 |
| EhTMKB1-11 | EHI_175920 | XM_643752 | XP_648844 |
| EhTMKB1-12 | EHI_001630 | XM_001914281 | XP_001914316 |
| EhTMKB1-14 | EHI_119250 | XM_645177 | XP_650269 |
| EhTMKB1-20 | EHI_123840 | XM_001914098 | XP_001914133 |
| EhTMKB1-24 | EHI_058930 | XM_001914144 | XP_001914179 |
| EhTMKB2-10 | EHI_117680 | XM_646485 | XP_651577 |
| EhTMKB2-11 | EHI_178610 | XM_651172 | XP_656264 |
| EhTMKB2-14 | EHI_068160 | XM_646638 | XP_651730 |
| EhTMKB2-15 | EHI_068610 | XM_001913383 | XP_001913418 |
| EhTMKB2-31 | EHI_180320 | XM_648738 | XP_653830 |
| EhTMKB2-36 | EHI_074740 | XM_649578 | XP_654670 |
| EhTMKB2-41 | EHI_064490 | XM_648927 | XP_654019 |
| EhTMKB2-74 | EHI_152420 | XM_651877 | XP_656969 |
| EhTMKB2-75 | EHI_092260 | XM_649463 | XP_654555 |
| EhTMKB2-77 | EHI_178610 | XM_651172 | XP_656264 |
| EhTMKB3-21 | EHI_124500 | XM_649751 | XP_654843 |
| EhTMKB3-28 | EHI_135730 | XM_648904 | XP_653996 |
| EhTMKB3-29 | EHI_050820 | XM_651538 | XP_656630 |
| EhTMKB3-32 | EHI_030120 | XM_648689 | XP_653781 |
| EhTMKB3-35 | EHI_158180 | XM_645723 | XP_650815 |
| EhTMKB3-37 | EHI_127280 | XM_647071 | XP_652163 |
| EhTMKB3-38 | EHI_110650 | XM_649171 | XP_654263 |
| EhTMKB3-42 | EHI_050480 | XM_651483 | XP_656575 |
| EhTMKB3-48 | EHI_127440 | XM_647225 | XP_652317 |
| EhTMKB3-51 | EHI_118410 | XM_644811 | XP_649903 |
| EhTMKB3-96 | EHI_167650 | XM_650501 | XP_655593 |
| EhTMKC-9 | EHI_117590 | XM_646499 | XP_651591 |
| EhTMKC-13 | EHI_025280 | XM_647846 | XP_652938 |
| EhTMKC-39 | EHI_037140 | XM_643895 | XP_648987 |
| EhTMKC-60 | EHI_138750 | XM_645045 | XP_650137 |
| EhTMKC-63 | EHI_087800 | XM_650854 | XP_655946 |
| EhTMKC-71 | EHI_030420 | XM_644725 | XP_649817 |
| EhTMKD1-3 | EHI_201270 | XM_646909 | XP_652001 |
| EhTMKD1-18 | EHI_067070 | XM_643756 | XP_648848 |
| EhTMKD1-40 | EHI_064500 | XM_648928 | XP_654020 |
| EhTMKD1-70 | EHI_189290 | XM_645928 | XP_651020 |
| EhTMKD1-79 | EHI_180150 | XM_645052 | XP_650144 |
| EhTMKD2-19 | EHI_081790 | XM_646939 | XP_652031 |
| EhTMKD2-27 | EHI_109950 | XM_647108 | XP_652200 |
| EhTMKD2-44 | EHI_127000 | XM_646458 | XP_651550 |
| EhTMKD2-46 | EHI_134750 | XM_647033 | XP_652125 |
| EhTMKD2-50 | EHI_137040 | XM_647501 | XP_652593 |
| EhTMKD2-57 | EHI_023050 | XM_648409 | XP_653501 |
| EhTMKD2-64 | EHI_086050 | XM_647991 | XP_653083 |
| EhTMKD2-82 | EHI_164380 | XM_643584 | XP_648676 |
| EhTMKD2-97 | EHI_082220 | XM_646828 | XP_651920 |
| EhTMKD2-98 | EHI_040850 | XM_643878 | XP_648970 |
| EhTMKE-22 | EHI_186990 | XM_651330 | XP_656422 |
| EhTMKE-54 | EHI_188110 | XM_648585 | XP_653677 |
| EhTMKE-66 | EHI_194480 | XM_649600 | XP_654692 |
| EhTMKF-34 | EHI_179840 | XM_650491 | XP_655583 |
| EhTMKF-45 | EHI_171310 | XM_644859 | XP_649951 |
| EhTMKF-59 | EHI_097640 | XM_644333 | XP_649425 |
| EhTMKF-80 | EHI_065500 | XM_643494 | XP_648586 |

***E. dispar* TMKs**

| **EdTMK** | **Pathema_ID** | **mRNA** | **Protein** |
| --- | --- | --- | --- |
| EdTMKA-4 | EDI_059530 | XM_001737102 | XP_001737154 |
| EdTMKA-17 | EDI_002690 | XM_001738231 | XP_001738283 |
| EdTMKA-52 | EDI_228480 | XM_001739050 | XP_001739102 |
| EdTMKA-53 | EDI_213720 | XM_001734488 | XP_001734540 |
| EdTMKA-55 | EDI_330800 | XM_001741604 | XP_001741656 |
| EdTMKA-61 | EDI_022990 | XM_001735407 | XP_001735459 |
| EdTMKA-69 | EDI_111110 | XM_001734130 | XP_001734182 |
| EdTMKA-72 | EDI_348890 | XM_001733950 | XP_001734002 |
| EdTMKA-85 | EDI_157490 | XM_001737231 | XP_001737283 |
| EdTMKB1-1 | EDI_050710 | XM_001741632 | XP_001741684 |
| EdTMKB1-4 | EDI_142140 | XM_001734278 | XP_001734330 |
| EdTMKB1-5 | EDI_321240 | XM_001734677 | XP_001734729 |
| EdTMKB1-7 | EDI_008100 | XM_001733921 | XP_001733973 |
| EdTMKB1-8 | EDI_163850 | XM_001740813 | XP_001740865 |
| EdTMKB1-10 | EDI_017140 | XM_001735347 | XP_001735399 |
| EdTMKB1-11 | EDI_202940 | XM_001741267 | XP_001741319 |
| EdTMKB1-12 | EDI_013290 | XM_001739659 | XP_001739711 |
| EdTMKB1-13 | EDI_280160 | XM_001741832 | XP_001741884 |
| EdTMKB1-15 | EDI_060810 | XM_001734162 | XP_001734214 |
| EdTMKB1-17B | EDI_034690 | XM_001739039 | XP_001739091 |
| EdTMKB1-19 | EDI_175570 | XM_001735786 | XP_001735838 |
| EdTMKB1-20 | EDI_163420 | XM_001741887 | XP_001741939 |
| EdTMKB1-21 | EDI_239740 | XM_001737287 | XP_001737339 |
| EdTMKB1-22 | EDI_300200 | XM_001735591 | XP_001735643 |
| EdTMKB1-24 | EDI_107670 | XM_001740414 | XP_001740466 |
| EdTMKB1-25 | EDI_344090 | XM_001738147 | XP_001738199 |
| EdTMKB1-26 | EDI_119580 | XM_001739919 | XP_001739971 |
| EdTMKB1-27 | EDI_081790 | XM_001737086 | XP_001737138 |
| EdTMKB1-30 | EDI_132510 | XM_001738431 | XP_001738483 |
| EdTMKB1-33 | EDI_046680 | XM_001738962 | XP_001739014 |
| EdTMKB1-38 | EDI_178780 | XM_001734221 | XP_001734273 |
| EdTMKB1-41 | EDI_254270 | XM_001741023 | XP_001741075 |
| EdTMKB2-11 | EDI_100570 | XM_001737423 | XP_001737475 |
| EdTMKB2-14 | EDI_343280 | XM_001741111 | XP_001741163 |
| EdTMKB2-31 | EDI_294780 | XM_001739622 | XP_001739674 |
| EdTMKB2-36 | EDI_044740 | XM_001737775 | XP_001737827 |
| EdTMKB2-74 | EDI_007840 | XM_001735941 | XP_001735993 |
| EdTMKB2-75 | EDI_153990 | XM_001733764 | XP_001733816 |
| EdTMKB2-94 | EDI_017280 | XM_001735361 | XP_001735413 |
| EdTMKB2-21 | EDI_023930 | XM_001737548 | XP_001737600 |
| EdTMKB2-28 | EDI_129420 | XM_001741351 | XP_001741403 |
| EdTMKB2-29 | EDI_058780 | XM_001737317 | XP_001737369 |
| EdTMKB2-35 | EDI_216880 | XM_001738754 | XP_001738806 |
| EdTMKB2-37 | EDI_147040 | XM_001741076 | XP_001741128 |
| EdTMKB2-38 | EDI_340570 | XM_001738254 | XP_001738306 |
| EdTMKB2-42 | EDI_187110 | XM_001739574 | XP_001739626 |
| EdTMKB2-48 | EDI_065080 | XM_001736331 | XP_001736383 |
| EdTMKB2-96 | EDI_165830 | XM_001738325 | XP_001738377 |
| EdTMKC-9 | EDI_029300 | XM_001733520 | XP_001733572 |
| EdTMKC-13 | EDI_075090 | XM_001739752 | XP_001739804 |
| EdTMKC-39 | EDI_284180 | XM_001733584 | XP_001733636 |
| EdTMKC-71 | EDI_084940 | XM_001733354 | XP_001733406 |
| EdTMKD1-3 | EDI_034450 | XM_001738353 | XP_001738405 |
| EdTMKD1-18 | EDI_105240 | XM_001734395 | XP_001734447 |
| EdTMKD1-40 | EDI_336370 | XM_001740763 | XP_001740815 |
| EdTMKD1-70 | EDI_277240 | XM_001737866 | XP_001737918 |
| EdTMKD1-79 | EDI_243130 | XM_001740260 | XP_001740312 |
| EdTMKD2-19 | EDI_172640 | XM_001738045 | XP_001738097 |
| EdTMKD2-44 | EDI_190540 | XM_001733791 | XP_001733843 |
| EdTMKD2-46 | EDI_339130 | XM_001734385 | XP_001734437 |
| EdTMKD2-50 | EDI_163100 | XM_001736035 | XP_001736087 |
| EdTMKD2-64 | EDI_249380 | XM_001734865 | XP_001734917 |
| EdTMKD2-82 | EDI_234560 | XM_001733604 | XP_001733656 |
| EdTMKD2-97 | EDI_303830 | XM_001740647 | XP_001740699 |
| EdTMKD2-98 | EDI_059790 | XM_001737107 | XP_001737159 |
| EdTMKE-22 | EDI_016610 | XM_001739706 | XP_001739758 |
| EdTMKE-54 | EDI_208860 | XM_001734461 | XP_001734513 |
| EdTMKE-66 | EDI_289530 | XM_001741931 | XP_001741983 |
| EdTMKF-34 | EDI_165220 | XM_001738297 | XP_001738349 |
| EdTMKF-45 | EDI_308360 | XM_001739805 | XP_001739857 |
| EdTMKF-59 | EDI_032590 | XM_001734578 | XP_001734630 |
| EdTMKF-80 | EDI_199060 | XM_001739464 | XP_001739516 |

***E. invadens*** TMKs

| **EiTMK** | **Pathema_ID** | **Contig no** | **Position** |
| --- | --- | --- | --- |
| EiTMKA-4 | EIN_168000 | AANW02000044 | 21533- 24725 |
| EiTMKA-17 | [EIN_249610](http://pathema.tigr.org/tigr-scripts/Entamoeba/shared/GenePage.cgi?locus=EIN_249610) | AANW02000073 | 4294-1062 |
| EiTMKA-23 | [EIN_199810](http://pathema.tigr.org/tigr-scripts/Entamoeba/shared/GenePage.cgi?locus=EIN_199810) | AANW02000082 | 33249-30054 |
| EiTMKA-53 | [EIN_109810](http://pathema.tigr.org/tigr-scripts/Entamoeba/shared/GenePage.cgi?locus=EIN_109810) | AANW02000272 | 5459-2265 |
| EiTMKA-55 | [EIN_028990](http://pathema.tigr.org/tigr-scripts/Entamoeba/shared/GenePage.cgi?locus=EIN_028990) | AANW02000223 | 16655-13508 |
| EiTMKA-61 | [EIN_135940](http://pathema.tigr.org/tigr-scripts/Entamoeba/shared/GenePage.cgi?locus=EIN_135940) | AANW02000096 | 26859-23546 |
| EiTMKA-65 | [EIN_015560](http://pathema.tigr.org/tigr-scripts/Entamoeba/shared/GenePage.cgi?locus=EIN_015560) | AANW02000837 | 3641-373 |
| EiTMKA-69 | [EIN_111340](http://pathema.tigr.org/tigr-scripts/Entamoeba/shared/GenePage.cgi?locus=EIN_111340) | AANW02000438 | 7465-11224 |
| EiTMKA-72 | [EIN_219430](http://pathema.tigr.org/tigr-scripts/Entamoeba/shared/GenePage.cgi?locus=EIN_219430) | AANW02000031 | 55195-51945 |
| EiTMKB1-1 | EIN_278750 | AANW02001860 | 2220-4361 |
| EiTMKB1-2 | EIN_140870 | AANW02003531  AANW02002329 | 42-1859  121-1321 |
| EiTMKB1-3 | EIN_132080 | AANW02000858 | 3155-7224 |
| EiTMKB1-4 | EIN_144640 | AANW02003528  AANW02001217 | 122-1876  839-2359 |
| EiTMKB1-5 | EIN_048750 | AANW02000178 | 28936-32984 |
| EiTMKB1-6 | EIN_087660 | AANW02001277 | 4153-8216 |
| EiTMKB1-7 | EIN_278290 | AANW02001737  AANW02003492 | 3259-59  1914-1103 |
| EiTMKB1-8 | EIN_151490 | AANW02000163 | 29740-33824 |
| EiTMKB1-9 | EIN_010650 | AANW02000198 | 16666-14280 |
| EiTMKB1-10 | EIN_089760 | AANW02000281 | 21750-17684 |
| EiTMKB1-11 | EIN_142310 | AANW02002927  AANW02000345 | 1188-1  1-1560 |
| EiTMKB2-2 | [EIN_043220](http://pathema.tigr.org/tigr-scripts/Entamoeba/shared/GenePage.cgi?locus=EIN_043220) | AANW02001527 | 659-4716 |
| EiTMKB2-11 | [EIN_098680](http://pathema.tigr.org/tigr-scripts/Entamoeba/shared/GenePage.cgi?locus=EIN_098680) | AANW02000267 | 8187-5489 |
| EiTMKB2-31 | [EIN_138400](http://pathema.tigr.org/tigr-scripts/Entamoeba/shared/GenePage.cgi?locus=EIN_138400) | AANW02000142 | 18228-22347 |
| EiTMKB2-92 | [EIN_038750](http://pathema.tigr.org/tigr-scripts/Entamoeba/shared/GenePage.cgi?locus=EIN_038750) | AANW02000047 | 51700-45199 |
| EiTMKB2-94 | [EIN_026980](http://pathema.tigr.org/tigr-scripts/Entamoeba/shared/GenePage.cgi?locus=EIN_026980) | AANW02000737 | 4758-8313 |
| EiTMKB3-51 | [EIN_277480](http://pathema.tigr.org/tigr-scripts/Entamoeba/shared/GenePage.cgi?locus=EIN_277480) | AANW02001769 | 5536-3000 |
| EiTMKC-9 | [EIN_219990](http://pathema.tigr.org/tigr-scripts/Entamoeba/shared/GenePage.cgi?locus=EIN_219990) | AANW02000024 | 84085-80835 |
| EiTMKC-13 | [EIN_134310](http://pathema.tigr.org/tigr-scripts/Entamoeba/shared/GenePage.cgi?locus=EIN_134310) | AANW02000303  AANW02003871 | 23014-25735  1576-1007 |
| EiTMKC-39 | [EIN_072510](http://pathema.tigr.org/tigr-scripts/Entamoeba/shared/GenePage.cgi?locus=EIN_072510) | AANW02000014 | 65160-68619 |
| EiTMKC-60 | [EIN_136260](http://pathema.tigr.org/tigr-scripts/Entamoeba/shared/GenePage.cgi?locus=EIN_136260) | AANW02000054 | 26769-23248 |
| EiTMKC-63 | [EIN_222800](http://pathema.tigr.org/tigr-scripts/Entamoeba/shared/GenePage.cgi?locus=EIN_222800) | AANW02000367 | 19190-22557 |
| EiTMKC-71 | [EIN_034170](http://pathema.tigr.org/tigr-scripts/Entamoeba/shared/GenePage.cgi?locus=EIN_034170) | AANW02000064 | 10957-14257 |
| EiTMKD1-3 | [EIN_072950](http://pathema.tigr.org/tigr-scripts/Entamoeba/shared/GenePage.cgi?locus=EIN_072950) | AANW02000089 | 7922-11679 |
| EiTMKD1-40 | [EIN_108610](http://pathema.tigr.org/tigr-scripts/Entamoeba/shared/GenePage.cgi?locus=EIN_108610) | AANW02000318 | 8381-3687 |
| EiTMKD1-79 | [EIN_124430](http://pathema.tigr.org/tigr-scripts/Entamoeba/shared/GenePage.cgi?locus=EIN_124430) | AANW02001377 | 85-3621 |
| EiTMKD2-19 | [EIN_033980](http://pathema.tigr.org/tigr-scripts/Entamoeba/shared/GenePage.cgi?locus=EIN_033980) | AANW02000716 | 11311-7462 |
| EiTMKD2-27 | [EIN_136890](http://pathema.tigr.org/tigr-scripts/Entamoeba/shared/GenePage.cgi?locus=EIN_136890) | AANW02001147 | 5930-2106 |
| EiTMKD2-44 | [EIN_112060](http://pathema.tigr.org/tigr-scripts/Entamoeba/shared/GenePage.cgi?locus=EIN_112060) | AANW02000028 | 59776-63255 |
| EiTMKD2-46 | [EIN_288390](http://pathema.tigr.org/tigr-scripts/Entamoeba/shared/GenePage.cgi?locus=EIN_288390) | AANW02000516 | 12447-8793 |
| EiTMKD2-64 | EIN_273970 | AANW02001596 | 15-3792 |
| EiTMKD2-82 | EIN_134960 | AANW02000045 | 37516-40840 |
| EiTMKD2-97 | [EIN_239890](http://pathema.tigr.org/tigr-scripts/Entamoeba/shared/GenePage.cgi?locus=EIN_239890) | AANW02000122 | 11051-15094 |
| EiTMKE-22 | [EIN_036170](http://pathema.tigr.org/tigr-scripts/Entamoeba/shared/GenePage.cgi?locus=EIN_036170) | AANW02000018 | 25911-28614 |
| EiTMKE-54 | [EIN_222310](http://pathema.tigr.org/tigr-scripts/Entamoeba/shared/GenePage.cgi?locus=EIN_222310) | AANW02001809 | 5324-2363 |
| EiTMKE-66 | [EIN_137400](http://pathema.tigr.org/tigr-scripts/Entamoeba/shared/GenePage.cgi?locus=EIN_137400) | AANW02000086 | 28155-30871 |
| EiTMKF-34 | [EIN_243410](http://pathema.tigr.org/tigr-scripts/Entamoeba/shared/GenePage.cgi?locus=EIN_243410) | AANW02000015 | 66553-64995 |
| EiTMKF-45 | [EIN_013240](http://pathema.tigr.org/tigr-scripts/Entamoeba/shared/GenePage.cgi?locus=EIN_013240) | AANW02001376 | 54-2020 |
| EiTMKF-59 | [EIN_109840](http://pathema.tigr.org/tigr-scripts/Entamoeba/shared/GenePage.cgi?locus=EIN_109840) | AANW02000272 | 11662-13787 |
| EiTMKF-80 | [EIN_013310](http://pathema.tigr.org/tigr-scripts/Entamoeba/shared/GenePage.cgi?locus=EIN_013310) | AANW02000416 | 9496-11628 |
