# Supplementary material for: Serum-Dependent Selective Expression of EhTMKB1-9, a Member of Entamoeba histolytica B1 Family of Transmembrane Kinases
Source: PLoS Pathog. 2010 Jun 3;6(6):e1000929. doi: 10.1371/journal.ppat.1000929 (PMC2880585; doi:10.1371/journal.ppat.1000929)
Supplement: Table S4 — Comparison of kinase subdomains of EhTMKB1 members as per Hank's classification. (0.07 MB DOC) [file ppat.1000929.s007.doc]

**Table S4.** **Comparison of kinase subdomains of EhTMKB1 member as per Hank’s classification** (Hanks SK, Hunter T, 1995)**.**

| **Subdomains** | **I** | **II** | **III** | **IV** | **V** | **VIa** | **VIb** | **VII** | **VIII** | **IX** | **XI** | Kinase/  Pseudo^¶^ |
| --- | --- | --- | --- | --- | --- | --- | --- | --- | --- | --- | --- | --- |
| **Consensus sequence*** | GEGsFGIV | VAIK | EVAMLDK | GAVFIP | GSIQDIMNK | GISYLH | LHRDIKPDNFLVV | KLTDFGS | GTPKYMAPEVL | SDIYSFSITMLQiiTWQDPF | SWQQEPKERITI |
| **EhTMKB1-1** | GEGSFGIV | VAIK | EVAMLDK | GAVFIP | GSIQDIMNK | GISYLH | LHRDIKPDNFLVV | KLTDFGS | GSPIYMAPEVL | SDIYSFSITMLQiiTWQDPF | SWQQEPKERITI | Kinase |
| **EhTMKB1-2** | GEGSFGIV | VAIK | EVAMLDK | GAVFIP | GSIQDIMNK | GISYLH | LHRDIKPDNFLVV | KLTDFGS | GTPKYMAPEVL | SDIYSFSITMLQiiTWQDPF | SWQQEPKERITI | Kinase |
| **EhTMKB1-3** | GEGSFGIV | VAIK | EVAMLDK | GAVFIP | GSIQDIMNK | GISYLH | LHRDIKPDNFLVV | KLTDFGS | GTPKYMAPEVL | SDIYSFSITMLQiiTWQDPF | SWQQEPKERITI | Kinase |
| **EhTMKB1-4** | GEGSFGIV | VAIK | EVAMLDK | GAVFIP | GSIQDIMNK | GISYLH | LHRDIKPDNFLVV | KLTDFGS | GSPIYMAPEVL | SDIYSFSITMLQiiTWQDPF | SWQQEPKERITI | Kinase |
| **EhTMKB1-5** | GEGSFGIV | VAIK | EVAMLDK | GAVFIP | GSIQDIMNK | GISYLH | LHRDIKPDNFLVV | KLTDFGS | GTPKYMAPEIL | SDIYSFSITMLQiiTWQDPF | SWQQEPKERITI | Kinase |
| **EhTMKB1-6** | GEGSFGVV | VAIK | EVAMLDK | GAVFIP | GSIQDIMNK | GISYLH | LHRDIKPDNFLVV | KLTDFGS | GTPKYMAPE-- | - | - | Pseudo |
| **EhTMKB1-7** | GEGSFGIV | VAIK | EVAMLDK | GAVFIP | GSIQDIMNK | GISYLH | LHRDIKPDNFLVV | KLTDFGS | GTPKYMAPEVL | SDIYSFSITMLQiiTWQDPF | SWQQEPKERITI | Kinase |
| **EhTMKB1-8B** | GEGTFGIV | VAIK | EVAMLDK | GAVFIP | GSIQDIMNK | GISYLH | LHRDIKPDNFLVV | KLTDFGS | GTPIYTVQ----- | - | - | Pseudo |
| **EhTMKB1-9** | GEGTFGIV | VAIK | EVAMLDK | GAVFIP | GSIQDIMNK | GISYLH | LHRDIKPANFLVV | KLTDFGS | GTPKYMAPEIL | SDIYSFSITMLQiiTWQDPF | SWEQEPKERITI | Kinase |
| **EhTMKB1-10** | GEGSFGIV | VAIK | EVAMLDK | GAVFIP | GSIQDIMNK | GISYLH | LHRDIKPDNFLVV | KLTDFGS | GTPKYMAPEVL | SDIYSFSITMLQiiTWQDPF | SWQQEPNKRLTT | Kinase |
| **EhTMKB1-11** | GEGTFGIV | VAIK | EVAMLDK | GAVFIP | GSIQNIMNK | GISYLH | LHRDIKPDNFLVV | KLTDFGS | GTPKYMAPEIL | SDIYSFSITMLQiiTWQDPF | SLQQESKERITI | Kinase |
| **EhTMKB1-12** | GEGSFGVV | VAIK | EVAMLDK | GAVFIP | GSIQDIMNK | GISYLH | LHRDIKPDNFLVV | KLTDFGS | GSPIYMAPEVL | SDIYSFSITMLQiiTWQDPF | SWQQEPKERITI | Kinase |
| **EhTMKB1-13** | GEGPFRVV | VAIK | EVAMLDK | GAVFIP | GSIQNIINK | GISYLH | LHRDIKPDNFLVV | KLTDFGS | GTPKYMAPEVL | SDIYSFSIIILQFITYFFFH | - | Pseudo |
| **EhTMKB1-14** | GEGTFGIV | VAIK | EVAMLDK | GAVFIP | GSIQDIMNK | GISYLH | LHRDIKPDNFLVV | KLTDFGS | GTPKYMAPEVL | SDIYSFSITMLQiiTWQDPF | SWQQEPKERITI | Kinase |
| **EhTMKB1-15** | GEGPFGIV | VAIK | EVAMIDK | GAVFIP | GSIQDITNK | GISYLH | LHRDIKPDNFLVV | KLTDFGS | GTPKYMAPEVL | SDIYSFSIIILQFITYFFFH | - | Pseudo |
| **EhTMKB1-16** | GEGPFGVV | VAIK | EVAMIDK | GTVFIP | GSIQDIMNK | GISYLH | LHRDIKPDNFLVV | KLTDFGS | GTPKYMAPEVL | SDIS | - | Pseudo |
| **EhTMKB1-17B** | GEGSFGVV | VAIK | EVAMLDK | GAVFIP | GSIQDIMNK | GISYLH | LHRDIKPDNFLVV | KLTDFGS | - | SDIYSFSVTMLQTITWQDPF | SWQQEPKERITI | Pseudo |
| **EhTMKB1-18A** | GEGTFGIV | VAIK | EVAMLDK | GAVFIP | GSIQDIMNK | - | - | - | - | - | - | Pseudo |
| **EhTMKB1-19** | GEGTFGIV | VAIK | EVAMLDK | GAVFIP | GSIQDIMNK | GISYLH | - | - | - | - | - | Pseudo |
| **EhTMKB1-20** | GEGSFGIV | VAIK | EVAMLDK | GAVFIP | GSIQDIMNK | GISYLH | LHRDIKPDNFLVV | KLTDFGS | GTPKYMAPEVL | SDIYSFSITMLQiiTWQDPF | SWQQEPKERIRI | Kinase |
| **EhTMKB1-21** | GEGTFGIV | VAIK | EVAMLDK | GAVFIP | GSIQDIMNK | GISYLH | - | - | - | - | - | Pseudo |
| **EhTMKB1-22** | - | - | EVAMLDK | GAVFIP | GSIQDIMNK | GISYLH | LHRDIKPDNFLVV | KLTDFGS | GTPKYMAPEVL | SDIYSFSITMLQiiTWQDPF | - | Pseudo |
| **EhTMKB1-23** | GEGIFGIV | VAIK | EVAMLDK | GGVFIP | GSIQNIMNK | - | - | - | - | - | - | Pseudo |
| **EhTMKB1-24** | GEGSFGVV | VAIK | EVAMLDK | GAVFIP | GSIQDIMNK | GISYLH | LHRDIKPDNFLVV | KLTDFGS | GTPKYMAPEVL | SDIYSFSITMLQiiTWQDPF | SWEQEPKERITI | Kinase |
| **EhTMKB1-25** | GEGSFGIV | VVIK | EVAMLDK | GALFIP | GSLQDIMNK | - | - | - | - | - | - | Pseudo |
| **EhTMKB1-26** | GEGSFGIV | VAIK | EVAMLDK | GTVFIP | GSIQDIMNK | GISYLH | LHRDIKPDNFLVV | KLTDFGS | - | - | - | Pseudo |
| **EhTMKB1-27** | - | - | - | - | - | - | - | - | ETPKYMAPEVL | SDIYSFSITMLQIITWQDPF | SWQQELNKRLITI | Pseudo |
| **EhTMKB1-28** | - | - | EVAMLDK | GAVFIP | GSIQDIMNK | _ISYLH | LHRDIKPDNFHVV | KLMDFES | - | - | - | Pseudo |

*- consensus sequence of EhTMKB1s as described by Beck *et al* 2005.

¶ -Degenerate, truncated kinases or absence of the conserved domain/residue classify kinase as pseudokinase (Manning *et al* 2002).
